# Exploring the landscape of current in vitro and in vivo models and their relevance for targeted radionuclide theranostics

**DOI:** 10.1007/s00259-025-07123-3

**Published:** 2025-02-28

**Authors:** Lisa Bokhout, Joana D. Campeiro, Simone U. Dalm

**Affiliations:** Department of Radiology and Nuclear Medicine, Erasmus MC, Rotterdam, The Netherlands

**Keywords:** Targeted radionuclide theranostics, In vitro models, In vivo models, Preclinical research, Cancer

## Abstract

Cancer remains a leading cause of mortality globally, driving ongoing research into innovative treatment strategies. Preclinical research forms the base for developing these novel treatments, using both in vitro and in vivo model systems that are, ideally, as clinically representative as possible. Emerging as a promising approach for cancer management, targeted radionuclide theranostics (TRT) uses radiotracers to deliver (cytotoxic) radionuclides specifically to cancer cells. Since the field is relatively new, more advanced preclinical models are not yet regularly applied in TRT research. This narrative review examines the currently applied in vitro, ex vivo and in vivo models for oncological research, discusses if and how these models are now applied for TRT studies, and whether not yet applied models can be of benefit for the field. A selection of different models is discussed, ranging from in vitro two-dimensional (2D) and three-dimensional (3D) cell models, including spheroids, organoids and tissue slice cultures, to in vivo mouse cancer models, such as cellline-derived models, patient-derived xenograft models and humanized models. Each of the models has advantages and limitations for studying human cancer biology, radiopharmaceutical assessment and treatment efficacy. Overall, there is a need to apply more advanced models in TRT research that better address specific TRT phenomena, such as crossfire and abscopal effects, to enhance the clinical relevance and effectiveness of preclinical TRT evaluations.

## Introduction

Cancer remains a major health problem worldwide; the disease is still a leading cause of death, with 9.7 million global deaths reported in 2022 [1]. Moreover, its incidence and mortality rate are expected to rise in the coming years [1, 2]. Logically, research into novel methods for early detection and treatment of the disease is constantly ongoing, both being crucial for disease prognosis. This includes the development of personalised treatment strategies, in which cancer treatment is tailored to the individual patient based on, for example, molecular expression patterns, with the ultimate goal to improve therapy response. In recent years, this has led to novel and improved interventions, including targeted radionuclide theranostics (TRT). TRT concerns the specific delivery of radionuclides to cancer cells using radiotracers targeted towards biomarkers that are (over)expressed on these cancer cells. A radiotracer consists of a high-affinity targeting agent (e.g. an antibody, a nanobody or a chemically synthesised peptide analogue that binds to the biomarker of interest) that is linked directly or via a spacer/linker to a chelator (i.e. a chemical compound that stably complexes a radionuclide). Radiotracers can be used for both (diagnostic) imaging and therapeutic purposes, hence the term theranostics, depending on the radionuclide applied. Radionuclides that emit γ rays and/or positrons can be used for single-photon emission computed tomography (SPECT) and/or positron emission tomography (PET) imaging, respectively. Radionuclides with higher energy, i.e. α- and/or β^-^-emitting radionuclides, are suitable for therapeutic purposes. Upon binding to its target, a radiotracer either remains at the cell membrane or is internalised by the cancer cell, causing accumulation of radiation at the cancer site, which can be detected with imaging or which eliminates the cancer cells. One advantage of TRT compared to more conventional radiotherapy, i.e. external beam radiotherapy (EBRT), is the specific delivery of radiation to cancer cells, allowing treatment of (small) metastases while limiting the damage to healthy tissues [3]. Additionally, compared to EBRT and other targeted therapies, radiotracers offer a dual advantage by enabling both imaging and therapy. This dual functionality allows for non-invasive patient selection based on target expression and facilitates ongoing monitoring of treatment response [4]. Furthermore, TRT-specific phenomena such as crossfire effects (e.g. the indirect irradiation of neighbouring cells by targeted radiotracers bound to adjacent cells) and bystander effects (e.g. the biological change in non-irradiated cells) can cause damage to (adjacent) tumour cells that lack biomarker expression, which is not feasible or less obvious with other targeted therapies [5]. The extent of crossfire effects is radionuclide dependent, i.e. the effect is much larger for β^−^-particles, which have a radiation range of 200–1000 cell diameters, than for α-particles with a range of 5–10 cell diameters [6, 7]. Moreover, the damaging effect of crossfire radiation is lower at larger distances due to the radiation inverse square law. Both direct and indirect irradiation lead to DNA single and/or double-strand breaks, the generation of reactive oxygen species and reactive nitrogen species, all potentially leading to cancer cell death [5, 8].

In 1994, the FDA approved [^111^In]In-pentetreotide (OctreoScan™) as the first somatostatin receptor subtype 2 (SSTR2) targeting radiotracer for SPECT imaging of SSTR2-positive neuroendocrine tumours (NETs) [9]. In subsequent years, several SSTR2-targeted radiotracers for PET imaging were developed, including [^68^Ga]Ga-DOTATATE (Netspot^®^), which received FDA approval in 2016 [10]. This was swiftly followed by the approval of the first SSTR2-targeted therapeutic radiotracer [^177^Lu]Lu-DOTATATE (Lutathera^®^) in 2018 for the treatment of gastrointestinal tract and pancreas NETs [11]. Later, in 2020, the first prostate-specific membrane antigen (PSMA) targeted radiotracer [^68^Ga]Ga-PSMA-11 was approved for PET imaging in prostate cancer (PCa) patients, followed by the approval of the first PSMA-targeted therapeutic radiotracer [^177^Lu]Lu-PSMA-617 (Pluctivo^®^) in 2022 for the treatment of positive metastatic castration-resistant PCa patients [12, 13]. The approval of these radiotracers has positively impacted the field. As a response, novel targeted radiotracers are being developed that are directed towards other biomarkers, such as the fibroblast activation protein (FAP) expressed on cancer-associated fibroblasts in the tumour stroma of many epithelial cancers [14, 15], the gastrin-releasing peptide receptor (GRPR) overexpressed in breast cancer (BC) and PCa [16–20] and C-X-C chemokine receptor type 4 (CXCR4) overexpressed in multiple cancers including, pancreatic cancer (PC), BC and PCa [19].

Clinically relevant models are crucial for reliable results during the development of targeted radiotracers. Ideally, a research model forms a replica of the subject to be studied, where the required complexity of the model aligns with the research question that needs to be answered. The most used in vitro models in many research fields, including that of TRT, are two-dimensional (2D) cell cultures that either represent the cancer of interest and/or express the target(s) of interest. These models have recently been complemented by more complex in vitro models, such as spheroids, organoids and cultures on microfluidic chip systems. Similarly, more advanced options for in vivo research models, including humanized mice, are now used besides the commonly used murine models, e.g. mice inoculated with (human) cancer cells.

Unfortunately, advanced preclinical models are only sporadically used to study targeted radiotracers. In this narrative review, we discuss the preclinical models currently applied in TRT research, including their advantages and limitations, and debate the potential value of using not yet or only sporadically used advanced preclinical models in this area of research.

## In vitro research models

**Fig. 1:**
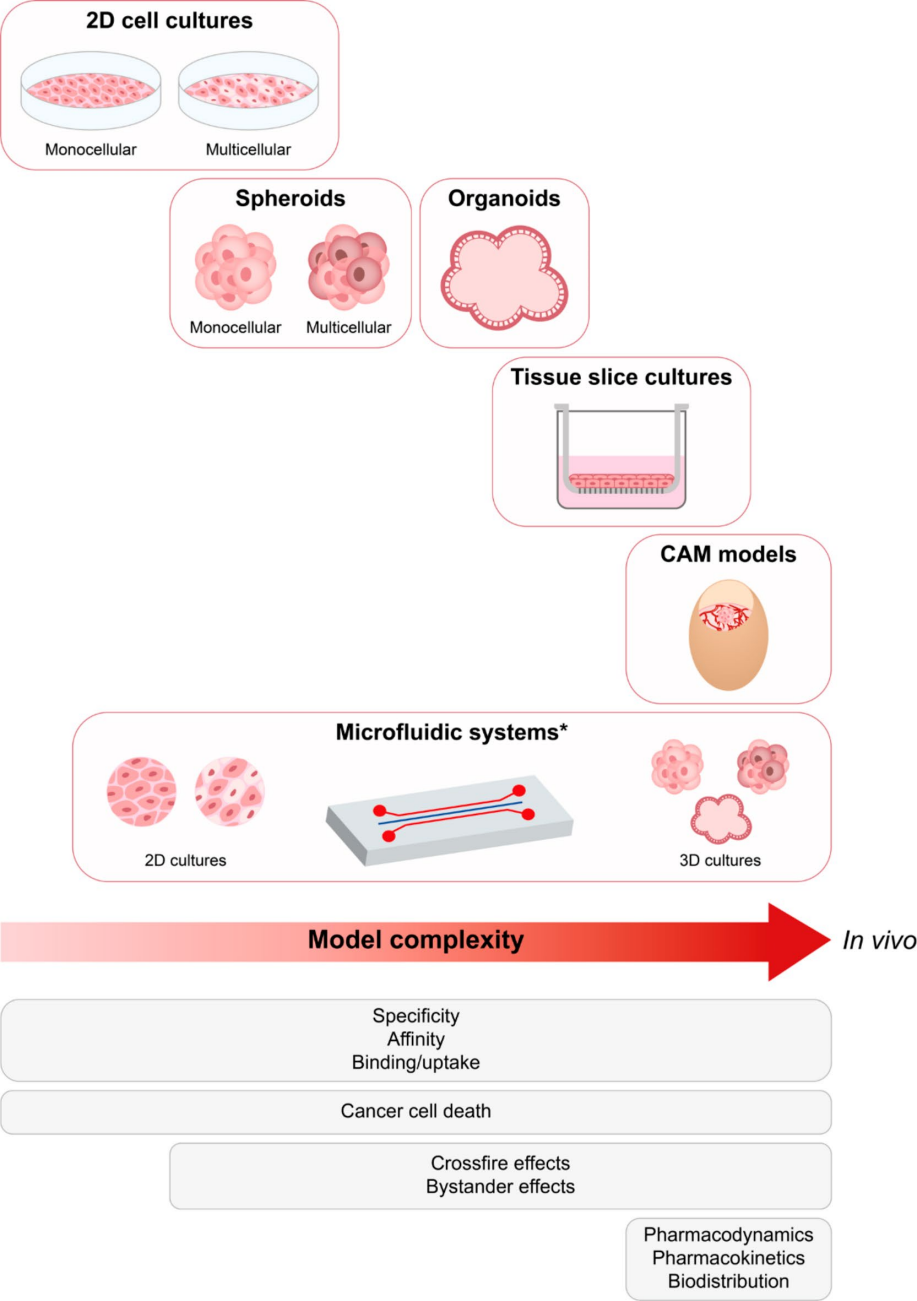
In vitro research models used in oncology research relevant for TRT research. The models are arranged from left to right in order of increasing complexity. Below, the properties and effects of radiotracers that can be evaluated in the corresponding models are listed.*Microfluidic systems vary in complexity based on factors such as the presence of multiple chambers representing different organs, or the type of cell models used in the system. CAM: chorioallantoic membrane

Experiments in in vitro models form the first step during the preclinical evaluation of diagnostic and therapeutic radiotracers. A variety of different in vitro models can be used, ranging from simple 2D cell cultures to more complex spheroids, organoids and tissue cultures. Such models differ in complexity, and each model has its own advantages and disadvantages. Figure 1 provides an overview of the in vitro models relevant for TRT research that are discussed below.

### 2D cell culture models

2D cell cultures are the most used and accessible in vitro research model. Such cell cultures can be generated using cell lines that are either finite or continuous. All cell lines are derived from primary cells isolated from tissue samples that divide a limited number of times before they become senescent. However, genetic alterations that occur naturally or are purposefully induced can immortalise cells, allowing continuous culture of cells as monolayers. In general, 2D cell cultures form an affordable, easy and high-throughput research model. Additionally, cell lines can be easily genetically modified by, for example, transforming or transducing the cell line to express a molecule of interest or to become fluorescent or bioluminescent (i.e. genetically modified cell lines (GMCLs)).

2D cell cultures have a broad range of applications in TRT research, including affinity studies, uptake studies, distribution studies and efficacy studies. For these experiments, cell lines with either endogenous expression of the target molecule(s) or GMCLs are required.

If GMCLs are used, a few factors need to be considered. First, target expression in GMCLs is often much higher than in cell lines with endogenous target expression [21]. This significantly higher expression can lead to an overestimation of the potential of the evaluated radiotracer. Moreover, genetic modification can cause abnormal protein localisation/accumulation in cellular compartments, abnormal protein function and can change the expression of other proteins, potentially causing cellular stress and altered cell behaviour. Furthermore, GMCLs generated in different labs can be genetically different due to, for example, random integration of the gene of interest. This could potentially disrupt other genes and lead to altered cell behaviour, which can (in)directly influence the therapy response of GMCLs generated in different labs [21]. The above-mentioned factors can influence and bias experiments and make it challenging to compare the results of studies performed in different laboratories. Such bias and misinterpretation can be prevented by validating and assessing the effect of the cell line modification on a genomic level, protein level, and in terms of functionality.

Apart from the above, the use of 2D cell cultures has limitations independent of genetic modification. First, the continuous long-term culture of cell lines using different culture conditions can lead to unintentional selection of cells that proliferate well in these culture conditions [22]. This ultimately changes the genetic profile of the cell line which might impact target expression levels and radiosensitivity. In a recent paper, Ben-David et al. compared the genetic variation across 27 strains of the BC cell line MCF7 cultured in various laboratories under diverse culture conditions [23]. Here, a large genetic variation was observed between the different strains, affecting gene expression and ultimately causing variable drug sensitivity and response [23].

Second, continuous cell lines cannot be generated from all tissues or tumours; in particular, difficulties arise with slow-growing tumours or tumours from non-proliferative tissues, e.g. NETs, sarcomas and/or gliomas, and cell lines with multiple target expression patterns [24]. In such cases, primary cells isolated from tissues are either non-proliferative by nature, fail to adhere to culture flasks or lose their proliferative abilities during sub-cultivation, limiting the number of available models for studying these cancers [25]. Regarding NETs, the scarcity of relevant cell lines is partly due to their low proliferation rates, which makes it challenging to establish continuous cell lines [26]. Accordingly, there are few relevant SSTR2-expressing NET cell lines available. Among these, the pancreatic NET cell lines BON1 and QGP-1, the small intestine NET cell line GOT1 and the lung NET cell line NCI-H727 are commonly used, but most of these cell lines have limitations. For one, despite most NETs having abundant SSTR2 expression, BON1 and QGP-1 have low SSTR2 levels [27]. As a consequence, cell lines with higher SSTR2 expression, such as the small cell lung cancer cell line NCI-H69 [28], are often used for SSTR2-directed TRT studies. A second example is the GOT1 cell line, which has a doubling time of more than 18 days [29], making this model extremely impractical to work with. Furthermore, questions have been raised regarding the relevance and authenticity of established NET cell lines due to limited knowledge of their origin and genomic characteristics [26]. For example, Boora et al. analysed the genome of six different NET cell lines and found that both BON1 and QGP-1 cells lack important genetic mutations that clinically characterise pancreatic NETs [30]. Recent developments, such as conditional reprogramming (CR), can support the generation of new cell models for tumours that are difficult to establish as immortalised cell lines. Conditionally reprogrammed cells (CRCs) are generated using tissue-derived primary cells that are co-cultured with irradiated fibroblasts. These fibroblasts provide nutrients while cell death is inhibited by adding a Rho kinase inhibitor to the cell culture medium to generate reversible “immortalised” cell cultures [31]. CR increases the success rate of maintaining long-term primary cell cultures [32], and using CR, many primary cell culture models have been formed from tissues, including PCa, BC, PC and healthy breast tissue samples [33–36]. Despite this, it should be emphasised that CR cannot be used to establish permanently immortalised cell lines. To our knowledge, CRCs have not been used for TRT studies yet. However, with the growing interest in TRT, CR might be of use to generate primary long-term cell culture models for cancers expressing a biomarker of interest for which few or no continuous cell lines exist. Of note, CR can also be a valuable tool to establish cell models that better replicate the biology of primary tumours, as CRCs better retain their original tumour cell morphology and are less prone to genetic drift compared to immortalised cell lines [32].

Third, 2D cell cultures do not accurately represent tissue structures, as they lack cell diversity and cell-cell contact. To a certain extent, this can be circumvented by co-culturing cancer cells with non-cancerous cells present in the tumour stroma. Such co-cultures can be a great option for in vitro evaluation of the crossfire effect of radiotracers on cellular components in the tumour microenvironment (TME) or to study the effect of the TME on TRT response. Although co-cultures are a relatively easy method to introduce cellular diversity in an experiment, there is little literature available on the use of co-cultures in the field of TRT [37–39]. For example, a co-culture containing both CAFs and cancer cells could be used in preclinical studies evaluating the potential of FAP-targeting radiotracers. These radiotracers bind to FAP on CAFs and potentially eliminate cancer cells by irradiating the CAFs or through the crossfire effect on adjacent cancer cells. Thus, a co-culture containing both CAFs and cancer cells is more representative and forms a more reliable model compared to FAP-overexpressing cancer cell lines that are now often used [40]. Unfortunately, co-cultures can be complicated to maintain due to differences in cell growth rates of the used cell lines, which can also cause batch-to-batch variability and complicate reproducibility. Moreover, further analysis of cells in co-cultures after radiotracer exposure can be challenging because relatively complex techniques are required to distinguish between the different cell types.

Although co-cultures are superior to 2D cell cultures when it comes to cellular heterogeneity, the tissue morphology is still not replicated because there is no extracellular matrix (ECM). The ECM interacts with cells in tissues (e.g. through receptor-ligand binding and signalling molecules), which is known to be important for cell proliferation, differentiation, gene expression and drug metabolism. This can thus impact the effect of and response to radiotracer treatment [41]. More advanced three-dimensional (3D) models can provide an answer to this.

### 3D culture models

#### Spheroids

The simplest 3D cell models are spheroids formed from aggregated cells that organise into sphere-like structures during cell proliferation and share metabolic similarities to their native tissues [42]. Spheroids can be homotypic (i.e. one cell type) or heterotypic (i.e. different cell types) and are cultured through various scaffold-free and scaffold-based techniques to induce cell aggregation [43]. Scaffolds such as (non-adhesive) hydrogels can guide the formation of spheroids, which induces more cell-cell and cell-matrix interactions that enhance spheroid formation [44]. Logically, heterotypic spheroids replicate solid tumours more accurately than homotypic spheroids because of their cellular heterogeneity, which also increases cellular signalling [45]. Of note, the cellular organisation of spheroids can mimic the nutrient- and oxygen-rich proliferating cell border and the necrotic, hypoxic and acidic core observed in solid tumours [45].

Spheroids form a valuable model for evaluating the penetration depth, cytotoxicity and therapeutic effects, including crossfire effects of diagnostic and/or therapeutic radiotracers due to their spherical nature [7, 46]. Moreover, the previously mentioned hypoxic environment that frequently exists in solid tumours can hinder the delivery of radiotracers. This impacts imaging accuracy and can reduce the effect of radionuclide therapy because less reactive free radicals are formed in the absence of oxygen [47]. Moreover, the absence of the oxygen effect (i.e. increased radiosensitivity due to the presence of oxygen) results in less DNA damage and higher radiation doses are required to reach an optimal therapeutic effect. The radiosensitizing effect of oxygen, expressed as oxygen-enhancement ratio (OER), varies for different LET radiations. The effect decreases with increasing LET radiations, which means that the therapeutic effect of high LET radiations (e.g. α-emitters) is not influenced by oxygen [48]. This makes targeted α-particle therapy a valuable option for the treatment of hypoxic tumours. Accordingly, hypoxic spheroids, similar to hypoxic tumours, require higher radiation doses to achieve the same level of DNA damage as normoxic spheroids or 2D cell cultures [49, 50]. Taken together, spheroids form a representative model that can provide more accurate outcomes when studying TRT efficacy in hypoxic tumours.

PC spheroids were used as early as 2006 by Wang et al. to test the cytotoxic effect of targeted α-particle therapy using ^213^Bi-labelled immunoconjugates [51]. More than a decade later, the use of spheroids in the TRT field has become more common. Spheroids formed from a variety of different cancer cell lines, using either scaffold-free or scaffold-based culture methods, were used to evaluate novel radiotracer(s) (combinations) for PCa [51–54], osteosarcoma [55], colorectal cancer [56], neuroblastoma [57], meningioma [58], melanoma [59], NETs [60] and HER2-positive cancers [61, 62]. The spheroids were used for a wide range of experiments, including cytotoxicity studies and radiotracer distribution studies. Regarding cytotoxicity studies, the effects were either determined by assessing cell viability [52, 55, 56, 59, 63], DNA damage [62, 64], live/dead markers [55], spheroid size [51, 52, 55, 60, 64, 65], growth [54, 56, 57, 61], cell count [53] and/or disintegration [55, 61] or colony-forming abilities [59]. Radiotracer distribution was generally visualised using fluorescence microscopy [53, 62, 63], but micro-autoradiography was also applied [62]. Further molecular effects of radiotracers on spheroids were assessed by analysing gene and/or protein expression levels [56, 57, 59, 60, 66].

**Fig. 2:**
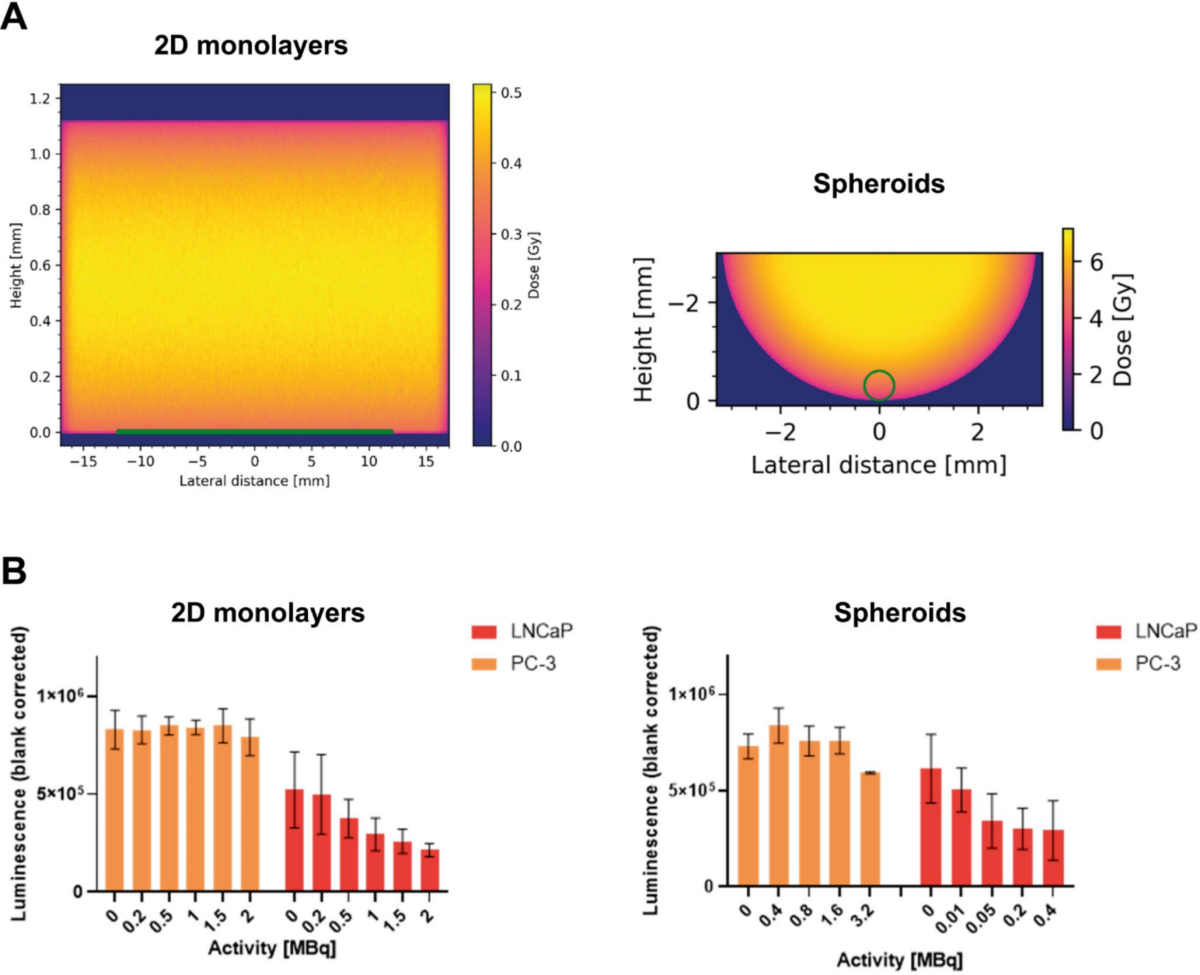
Comparison of targeted radionuclide therapy efficacy in 2D and 3D cell culture models. (**A**). Monte Carlo simulation of dose distribution after 3-hour treatment with an activity of 1 MBq. (**B**). Cell viability of the prostate cancer cell lines LNCaP and PC3 cultured in monolayers and spheroids, 7 days after 3-hour treatment with variable activities of [^177^Lu]Lu-PSMA-I&T. Reproduced under CC BY 4.0 license with permission from MDPI, Raitanen J., Barta B., Fuchs H., et al. Radiobiological Assessment of Targeted Radionuclide Therapy with [177Lu]Lu-PSMA-I&T in 2D vs. 3D Cell Culture Models. Int. J. Mol. Sci. 2023, 24(23), 17,015 [52]

When using spheroids for preclinical TRT research, it should be taken into consideration that there is a discrepancy in the literature on the effect of radiation on spheroids. Multiple studies report that spheroids are either more sensitive or more resistant to radiation compared to 2D cell cultures. In a study by Raitanen et al., Monte Carlo simulations have shown that the geometry of cell culture plates and the shape of the model (i.e. spherical or flat) influence how much ionising radiation is absorbed by the cells [52]. When cells cultured as monolayers and spheroids are treated under the same conditions, the models have a theoretically absorbed dose of 0.25 Gy and 4.54 Gy, respectively (Fig. 2A) [52]. Indeed, cell viability in monolayers was higher compared to cells cultured as spheroids, and LNCaP spheroids were 2.4-fold more sensitive to 0.2 MBq [^177^Lu]Lu-PSMA-I&T than cells cultured in monolayers (Fig. 2B) [52]. However, it has also been described that spheroids of the same cell line are more therapy-resistant compared to 2D cell cultures [67, 68]. This discrepancy could be explained by the spheroid morphology and organisation, in some cases leading to more hypoxic models, which, as aforementioned, are generally more radioresistant [49].

The size of a spheroid and the level of target protein expression can also play a role in radiotracer distribution [53, 63]. This could present challenges in larger spheroids, such as reduced penetration and uneven distribution of radiotracers due to increased cellular density within the spheroid. It is essential to consider these challenges when designing experiments and analysing data, especially when it is not representative for the tumour type of interest.

The enhanced complexity of spheroids compared to 2D cell cultures provides a more clinically representative model, but spheroid models can be low throughput, results can be difficult to reproduce, and studies can be labour intensive. Additionally, spheroids still lack an ECM that is important for in vivo-like cell behaviour and influences therapeutic responses [41].

#### Organoids

Organoids are self-renewable, multicellular 3D structures with a more complex organisation [69]. They are formed from cells with stem cell-like properties that aggregate and self-organise in a scaffold, often a natural or synthetic hydrogel. Organoids are produced from primary cells such as tissue-resident adult stem cells (AdSCs) that are found in (tumour)tissues, pluripotent stem cells (e.g. induced pluripotent stem cells (iPSCs)) and embryonic stem cells [70–72]. To generate organoids, single cells are plated in a hydrogel that replicates the *lamina propria* (i.e. the connective tissue that supports cells in tissues) to induce cell aggregation and form an ECM, allowing transmission of biochemical and biomechanical signals between cells. Cells are provided with growth factor-supplemented culture medium to deliver nutrients needed for cell proliferation and the generation of 3D cell masses [73]. Organoids present similar morphologies and functions to their native tissues, including hypoxia that is observed in solid tumours and the herewith associated radioresistance [69, 74, 75]. RNA-seq analysis has further confirmed the genetic resemblance of tumour-derived organoids [76].

To our knowledge, no studies have been published describing the use of organoids in the TRT field. However, organoids are used to study other targeted therapies and EBRT [74, 76–78]. Such studies share similarities with experiments now performed on 2D cell cultures and spheroids in TRT research, such as affinity, uptake and cytotoxicity studies. In the context of TRT, organoids can be highly relevant for diagnostic and therapeutic radiotracer evaluation, especially when it comes to distribution, therapy efficacy and the potential crossfire and bystander effect on cells in the TME. Although the latter two can be studied in 2D co-cultures to a certain extent, patient-derived organoids (PDOs), generated from either AdSCs or iPSCs, are more representative when it comes to clinical tumour composition (i.e. a larger variety in cellular components in the TME). Using PDOs can thus improve the translation of radiotracers from bench-to-bedside and provide valuable information by correlating the therapeutic response of these organoids to the phenotype and/or genotype of the tumour it is derived from.

It is, however, important to note that there is inter- and intra-batch-to-batch variability in PDO cultures, such as variations in growth rate, cell composition and genetic background, but this can be compensated for by including more biological replicates in experiments. However, the availability of patient material and the varying success rate of PDO cultures, depending on the tissue origin and variability in culture protocols used, form a major limitation [79, 80]. The lack of a standardised culture protocol further affects the reproducibility and comparability of different studies [70]. Novel innovations, such as bioprinting [81], can improve the reproducibility of organoid cultures, but these complex techniques are not widely available.

Of note, despite the superior biological complexity of organoids, there is still a lack of important biophysical factors, such as pressure and perfusion, that influence cellular response and behaviour, and inevitably therapeutic response [82]. However, biophysical factors can be applied to organoids by combining organoid culture with microfluidic systems (see paragraph 2.3).

#### Organotypic tissue slice cultures

Organotypic tissue slice cultures (TSCs) are used to maintain (human) tissue slices for in vitro experiments. For this, slices with a thickness of 250–500 μm are cut from either healthy organs or tumour samples within 6 hours after surgical removal and are maintained in cell culture medium. These slices contain all cells present in the native tissue and retain the original tissue structure and morphology, the latter allowing cell-cell interactions and chemical communication. Tissue slices can be cultured either scaffold-free, filter-supported or in a 3D tumour slice culture (3D-TSC) [83]. Using the scaffold-free and filter-supported culture method, tissue slices are maintained in cell culture medium in culture plates or dishes with or without a plate insert, whereas 3D-TSC uses collagen as a scaffold to preserve the shape and composition of the original tumour. TSCs cultured through either technique can be used for drug evaluation studies. Validation of the 3D-TSC model after 8 days of culture showed histological and transcriptome similarities when compared to the original tumour, as well as preservation of the TME [83].

No studies have reported the use of TSCs in the TRT field to our knowledge. However, it could be a relevant model because the TME in tumour TSCs allows evaluation of the crossfire and bystander effects of radiotracers. TSCs have been used in other related fields to study the therapeutic efficacy of small-molecule drugs, immunotherapy, and radiotherapy where head and neck squamous cell carcinoma tumour and organotypic brain TSCs were used to study the effects of proton beam radiotherapy (PBRT) [84]. The cell survival, metabolic activity and cell morphology of the tissue slices were determined before PBRT treatment. Histological analysis of tumour TSCs confirmed comparable cell proliferation and tissue morphology to the tumour of origin [84]. PBRT caused DNA double-strand breaks, identified by γH2AX staining, proving that these models are suitable to assess the effects of radiation therapy [84].

Unfortunately, TSCs also have drawbacks; they are difficult to maintain due to complex culture conditions, and if maintained, they have a limited lifespan, which restricts their suitability for long-term experiments.

### Microfluidic systems

Microfluidic systems are versatile cell culture devices that can be used to culture cells in constantly perfused chambers that mimic blood flow and other fluid dynamics in tissues and/or organs. Commonly used microfluidic systems include chips and nanowell plates that are formed through varying production methods, such as bioprinting and mould casting [85]. Microfluidic chips can be simplistic with only one cell type cultured in a single channel or have intricate designs where multiple channels are lined with different cell types, either 2D or 3D cell cultures, to replicate tissue structures or whole organs. These features allow the production of intricate and customisable in vitro models that incorporate the influence of perfusion and physical stress on cell behaviour [86].

The use of microfluidic systems for drug development started with the Shuler group, who have since produced multiple microfluidic devices to study the toxicity, distribution, adsorption, metabolism and elimination of drugs under the commercial name Hesperos Inc [87, 88]. Such systems have not yet been described in the TRT field, but they have potential for studying the pharmacodynamics (PD) and pharmacokinetics (PK) of diagnostic and therapeutic radiotracers. The PD and PK of radiotracers are currently assessed in mouse models that can have different target expression profiles in organs than humans do, which hinders clinical translation. Here, the use of microfluidic models might be more accurate as solely human cells are used. For such studies, a relatively complex model with multiple compartments (i.e. “organs”) should be applied, also known as body- or human-on-a-chip. These compartments should mimic the organ it represents as much as possible by, for example, designing multiple channels in the compartment that serve as endothelial barriers and blood vessels across which the radiotracer can transfer into the “tissue” [89]. Additionally, the different chip compartments (i.e. individual “organs”) should be connected with each other and fluid perfusion through the system should be generated by using, for example, a pump. When using organ-on-chips to determine the PD and PK of radiotracers, it should be considered that the model is still a simplified version of the human organ and/or body, and further in vivo validation may be required. Nevertheless, using such systems could reduce the number of animals used.

Microfluidic systems can also be applied in diagnostic and therapeutic radiotracer uptake and efficacy experiments to implement the effect of radiotracer circulation and distribution. This potentially provides a more accurate indication of in vivo tumour uptake and thus a more accurate determination of an optimal dose for (diagnostic) imaging and treatment for subsequent in vivo experiments, without prior use of animal models. Moreover, toxicity in (dose-limiting) tissues or organs could be studied using microfluidic systems, similar to other preclinical EBRT studies that used microfluidic chips to determine the effects of radiation on healthy cells and cancer cells, including spheroids [49]. One of these studies has shown that human umbilical vein endothelial cells (HUVECs) cultured as 3D tubes on microfluidic chips were more radioresistant compared to HUVECs cultured as monolayers, which were more prone to lose cell-cell contact and undergo apoptosis when exposed to higher radiation doses [90]. The degree of DNA damage was similar, but double-strand DNA breaks were repaired more rapidly in HUVECs cultured on microfluidic chips [90]. This study emphasises the importance of how cells are cultured and how different cell culture techniques can influence experimental outcomes, especially when the structure in which the cells are cultured differs from their native structure.

There are a few limitations and disadvantages of using microfluidic devices. First, culturing cells in microfluidic systems is very labour-intensive, time-consuming and it can be difficult compared to other culture methods. Additionally, this culture method is more prone to inter- and intra-batch-to-batch variability with increasing complexity of the system. More errors and variability are introduced if more complex microfluidic systems are used, which reduces the chance of producing consistent experimental output [91]. Moreover, since the use of microfluidic devices is relatively limited and new, there are no standardised protocols available yet and no official test criteria exist, which makes it complicated to compare and verify the reliability of different study outcomes [92]. This will only improve with increasing use of these systems, which might be stimulated by the recent change in the FDA guidelines that animal testing is no longer required for the approval of pharmaceuticals [93].

## Chorioallantoic membrane model

The chorioallantoic membrane (CAM) is a highly vascularised membrane in avian embryos that is used as a research model for cancer therapies. The CAM model is not considered an animal model when experiments are performed on embryos that have not yet hatched. Avian embryos develop the CAM between 4 and 10 days after fertilisation, which is xenografted, on average, between embryonic development day (EDD) 8 and 10 [94]. To do so, the chorioallantoic vein is located, and a small hole opposite of the vein is drilled in the eggshell to create an air chamber between the CAM and the eggshell. Another small hole is drilled near the vein to remove part of the eggshell to reveal the CAM, where cancer cells or tumour tissue can be implanted. In general, xenografts will not be rejected as the CAM is partially immune deficient. CAM models have various applications to study in ovo tumour characteristics and behaviour, such as metastases formation, angiogenesis, stemness potential and therapy response [95]. Furthermore, tumours in CAM models commonly form within 2 to 5 days after xenografting, which is on average much faster compared to other in vivo models [96]. Lastly, CAM models are cost-efficient and can bridge in vitro and in vivo mouse studies [95].

The CAM model has been used in studies focusing on different types of cancer, such as PCa [97–99], glioblastoma [100], PC [101] and head and neck cancer [102]. Yet, the use of the CAM model has not been described much in the TRT field. However, actions have been taken by a German research group to develop and test a functional CAM model for diagnostic and therapeutic radiotracer studies. In a proof-of-principle study by Winter et al. PCa CAM models were developed using PSMA-positive PCa cell lines LNCaP, LNCaP C4-2 and PSMA-negative PCa cell line PC3. Using this model, PET/magnetic resonance imaging (MRI) was performed between EDD12 and EDD16 using [^68^Ga]Ga-PSMA-11 to determine tumour uptake over time [99]. After imaging, tumours were collected to determine absolute tumour uptake ex vivo and immunohistochemical stainings were performed to verify PSMA expression. As expected, a significantly higher uptake of [^68^Ga]Ga-PSMA-11 was observed in PSMA-positive tumours compared to PSMA-negative tumours. These results were very similar and within the same order of magnitude as results obtained in studies examining PSMA-targeted radiotracers in mice. In a follow-up study by Löffler et al. the LNCaP C4-2 and PC3 PCa CAM models were further evaluated to determine if radiotracer blocking studies could be performed in CAM models with PET/MRI using [^18^F]siPSMA-14 [98]. Indeed, a clear inhibitor-dependent trend in tumour uptake of the radiotracer was observed in the presence of the PSMA inhibitor 2-PMPA. The abovementioned studies collectively indicate that CAM models could form an alternative model to xenograft mouse models for screening in vivo binding and tumour uptake of radiotracers.

The CAM model also has limitations. For one, experiments using this model are time restricted since experiments using avian embryos should be terminated before eggs hatch. In most cases, CAM models can only be used up until EDD18 at the latest because the embryos start developing a sense of pain around EDD15 [99]. CAM models are, therefore, unsuitable to study long-term (therapy) effects [103]. Furthermore, performing post-CAM experiments, such as immunohistological stainings, is hindered by a lack of suitable reagents for avian samples, which limits experimental options [96].

## In vivo research models

Despite the development of novel and more complex in vitro models, studies using living animals remain a crucial and necessary step for the clinical translation of novel drugs and therapy strategies. For one, in vitro studies can only provide limited evaluation of complex parameters such as PK, PD, biodistribution, and off-target organ toxicity, or determine the effects of TME components/characteristics on treatment efficacy. Parameters relevant for TRT, such as crossfire and bystander effects can be studied to a certain extent in more advanced 3D in vitro systems; however, there are limitations to these models as addressed above. Moreover, more complex models are required to study abscopal effects of TRT, i.e. the positive and/or negative effects of local tumour therapy on distant (cancer) tissues and/or metastases.

A variety of different animal models are used for preclinical research, including both mammalian and non-mammalian models. Even though large mammals have more genetic similarities to humans, especially pigs, dogs and non-human primates, mice are commonly used for cancer research. This is among others due to the availability of highly standardised inbred strains. Moreover, the use of mice has the advantages of convenient feeding, low costs and easy genetic modification [104]. Additionally, the mouse genome is highly homologous to the human genome, and therefore, mice undergo many similar biological processes, including the development and spreading of cancer [104, 105].

Depending on the research question, healthy and/or diseased animal models should be used. In some instances, drug characteristics such as PK, PD, drug stability, off-target organ uptake, and even safety of (new) cancer therapies should be assessed in healthy animals. On the other hand, studies evaluating aspects such as tumour uptake and efficacy of anti-cancer agents require representative disease models. Such models can be obtained through various methods, including cancer cell inoculation or tissue xenografting (i.e. the transplantation of cells or tissue from one species into another species), chemical induction, and genetic engineering.

**Fig. 3:**
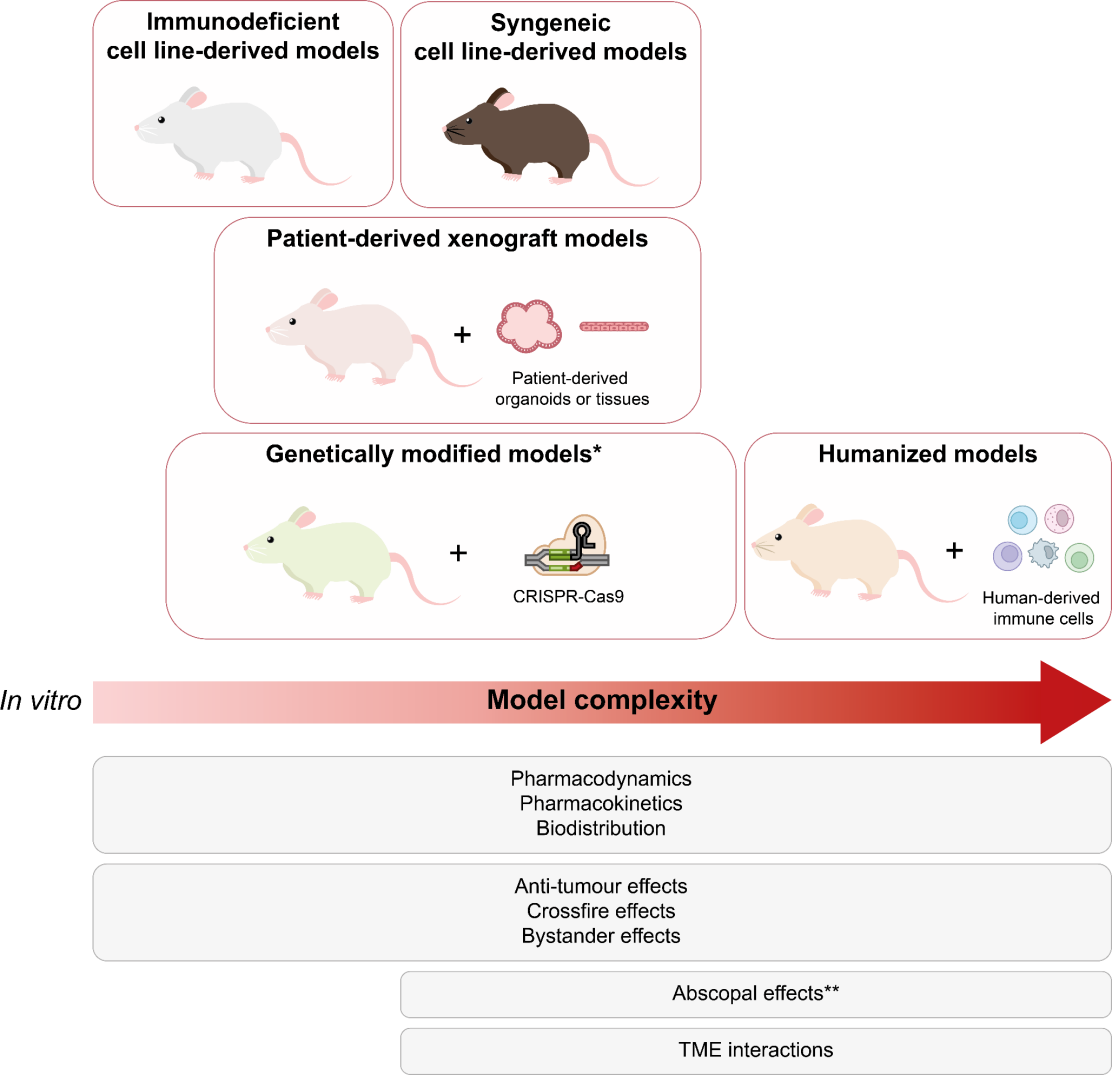
In vivo mouse models used in oncology research relevant for TRT research. The models are arranged from left to right in order of increasing complexity. Below, the properties and effects of radiotracers that can be evaluated in the corresponding models are listed. *Both immunodeficient and immunocompetent mice can be used to generate genetically modified mouse models. **Abscopal effects can only be studied in immunocompetent mice

In the field of TRT, murine cancer models are the most used in vivo model, and this part of the review will therefore be limited to these models. Figure 3 provides an overview of the mouse models that are discussed.

### Cell line-derived mouse models

In cell line-derived tumour models, mice are inoculated with cancer cells without or with a matrix-forming gel (to facilitate tumour formation). Typically, human cancer cells are xenografted in immunocompromised mice to prevent immune rejection. The cells are injected either under the skin (i.e. subcutaneous) or in the native organ the tumour forms in (i.e. orthotopic). Compared to subcutaneous tumours, orthotopic tumours provide a more realistic interaction between the tumour and the organ-specific microenvironment. In both cases, the tumour will commonly develop within 1 to 8 weeks depending on the number of cancer cells injected, the aggressiveness and/or tumourigenicity of the cell line and the in vivo growth rate. The size of subcutaneous tumours can easily be assessed using a calliper; however, it is more complicated to measure the size of orthotopic tumours. Tumourgrowth and metastatic spread can also be tracked using imaging techniques, such as ultrasound, PET/CT, MRI, and bioluminescent and fluorescent imaging [106, 107]. If optical imaging is used, mice should be inoculated with GMCLs that express luciferase or fluorescent proteins. However, it should also be noted that there are limitations to optical imaging. For one, bioluminescent or fluorescent signals might be too weak or scattered for detection when tumours or metastases are deeper within the body, as the light waves scatter while travelling through tissues before hitting the detector. Moreover, small tumours or micro metastases might not exceed the background signal and will therefore not be detected with bioluminescent imaging, since the emitted signal is dependent on the type of luciferase and the degree of expression [108]. Furthermore, high background signals caused by auto-fluorescent tissues, such as the albumin-rich liver, can hinder cancer cell detection. In general, cell line-derived tumour models are easy to use, relatively low in costs, reproducible and a sufficient number of established cell lines and mouse strains with known genetics are available.

**Fig. 4:**
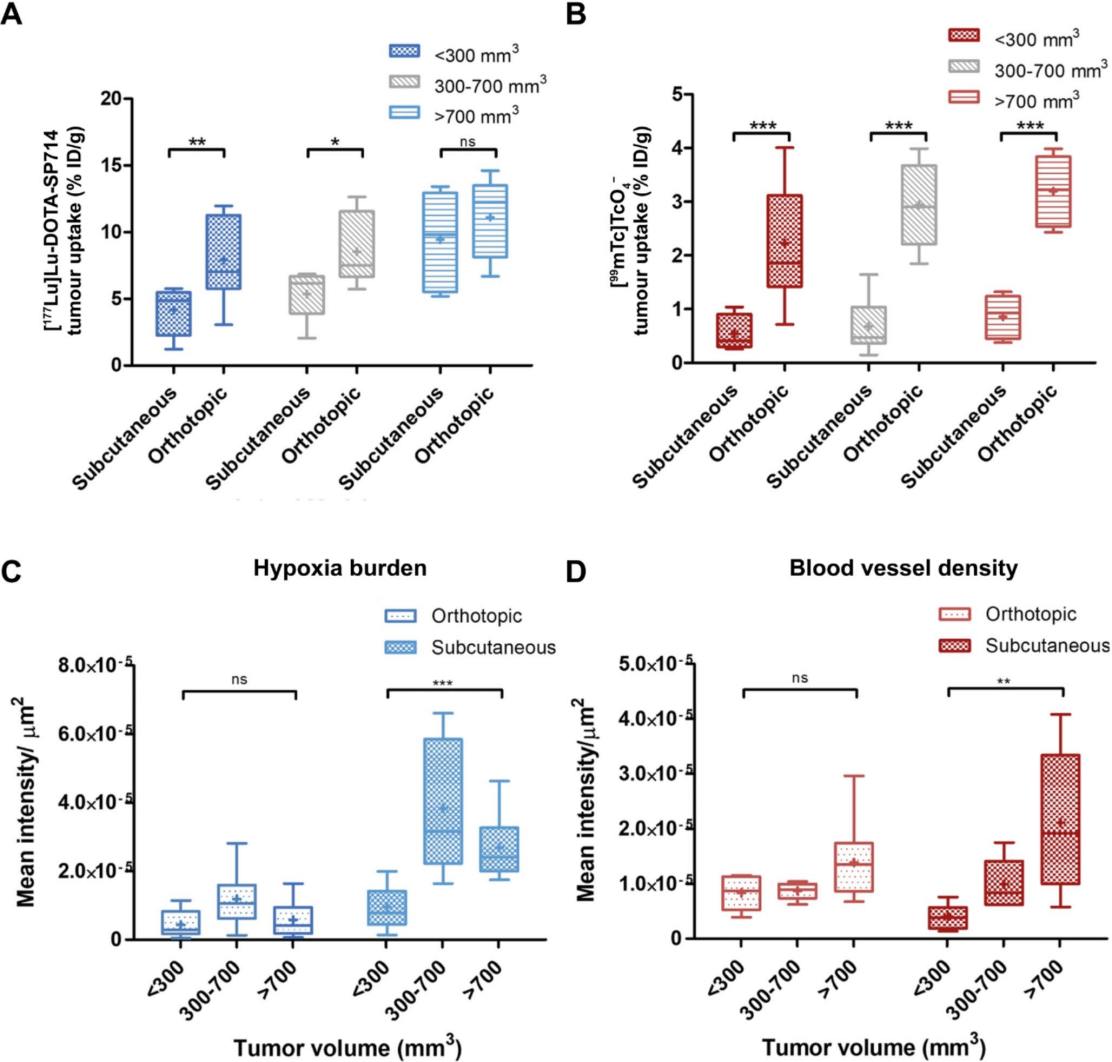
Comparison of characteristics of subcutaneous and orthotopic PC3 tumours in immunodeficient mice. (**A**). Tumour uptake of GRPR-targeting radiotracer [^177^Lu]Lu-DOTA-SP714. (**B**). Tumour uptake of [^99m^Tc]NaTcO4^-^ as a measure of tumour perfusion. (**C**). Ex vivo quantification of hypoxia burden and (**D**). blood vessel density of tumour slices. (**A-D**) are categorised based on tumour volume (mm^3^), +: mean, line at median. Reproduced under CC BY 4.0 license with permission from Springer Nature, Zhang, W., Fan, W., Rachagani, S. et al. Comparative Study of Subcutaneous and Orthotopic Mouse Models of Prostate Cancer: Vascular Perfusion, Vasculature Density, Hypoxic Burden and BB2r-Targeting Efficacy. Sci Rep 9, 11,117 (2019) [111]

At present, mice with subcutaneous tumours are the most used in vivo model for TRT research to study the biodistribution, tumour uptake and treatment efficacy of radiotracers [109, 110]. However, it can be beneficial to use orthotopic tumour models. In a study by Zhang et al. biological parameters of subcutaneous and orthotopic tumours were compared by xenografting mice with the human PCa cell line PC3. In both models, tumour distribution and uptake were determined using the GRPR-targeted radiotracer [^177^Lu]Lu-DOTA-SP714 (Fig. 4A). Tumour perfusion and microvasculature density were assessed using [^99m^Tc]NaTcO4^−^ (Fig. 4B) and Hoechst 33342 staining, and tumour hypoxia was measured using pimonidazole staining (Fig. 4C) [111]. Mice with orthotopic tumours demonstrated significantly higher levels of tumour perfusion compared to subcutaneous tumours, despite their similar vasculature. This could be explained by the higher interstitial fluid pressure in subcutaneous tumours, where the vasculature is denser (Fig. 4D) [111]. Furthermore, higher [^177^Lu]Lu-DOTA-SP714 and lower levels of hypoxia burden were observed in orthotopic tumours compared to subcutaneous tumours. Collectively, these results indicate that tumour perfusion plays an important role in radiotracer uptake, which should be considered when choosing a research model [111].

Despite their usefulness, cell line-derived tumour models have certain limitations. Firstly, tumours tend to be homogeneous when mice are inoculated with cells from a single cancer cell line, lacking the cell diversity and tissue structures observed in human tumours [112]. Heterogeneity in a tumour can easily be introduced by inoculating mice with multiple cancer cell lines. However, one of the cell lines might overgrow the other and cause a loss of tumour heterogeneity. Secondly, the small size of mice can make orthotopic inoculation challenging. This is especially the case for the inoculation of cells in small organ structures. In such cases, rats could serve as an alternative model. Due to their larger size, rats have somewhat bigger organ structures, which make surgical implantations of cells easier. However, it should be kept in mind that rats are not a direct substitute for mice [105]. Additionally, using immunocompromised mice as a model excludes the impact of the immune system on therapy response. Lastly, the TME has a different composition compared to human tumours due to the absence of immune cells and a lack of cell diversity. As an alternative, syngeneic mouse models can be used, where immunocompetent mice are inoculated with murine cancer cells. In these models, tumours develop under normal conditions in the presence of a functional immune system, similar to tumour formation in humans. Syngeneic models are thus a relatively easy model with increased in vivo complexity and a more representative TME; however, all these aspects are still fully murine. Unfortunately, the availability of murine cancer cell lines is limited, especially cell lines expressing targets relevant for TRT studies. Despite this, a few studies have described the use of syngeneic mouse models to evaluate TRT for multiple myeloma [113, 114], melanoma [59, 115] and triple-negative BC [116]. In these studies, either C57BL/6 or BALB/c immunocompetent mice were inoculated with murine cancer cells.

Despite the increased complexity of syngeneic models, these models are still not fully representative of human cancer because the tumours are not human-derived and mice lack aspects of human physiology, which potentially interferes with clinical translation [117]. More advanced models can overcome some of these limitations by, for example, using ‘humanized’ mice that are genetically modified to have a human-like immune system, as mentioned in paragraph 4.4. Furthermore, similarly to non-syngeneic models, the tumour formed in syngeneic mice remains homogeneous when a single murine cancer cell line is used, but this can be circumvented by inoculating a mix of different cancer cells as addressed above.

### Patient-derived xenograft mouse models

Patient-derived xenograft (PDX) mouse models are xenografted with patient-derived tumour tissues instead of cell line-derived cancer cells. In PDX models, small pieces of patient tumour tissues are implanted either subcutaneously, orthotopically or heterotypically (i.e. transplantation into a different location than the typical anatomical site) in immunocompromised or humanized mice [118]. After xenografting, it takes a few days to months before a tumour is formed, depending on the type of tumour tissue xenografted. Once a tumour of roughly 1–2 cm^3^ has formed, i.e. a first-generation tumour, the model is used for in vivo therapy studies or is passaged by resecting, sectioning and transplanting a piece of the tumour in other mice [118]. After xenografting, the tumours retain the morphology and genetic profile of the original tumour and exhibit a therapy response similar to that observed in the corresponding patients [119–123]. Overall, PDX models closely replicate patient tumours, making them relevant for personalised medicine. Thus, they provide valuable information that aids in clinical decision-making, ultimately enhancing therapeutic success.

Only a few papers describe the use of PDX models in the field of TRT, focusing on PSMA-positive and PSMA-negative PCa [124], non-small cell lung cancer (NSCLC) [125] and acute lymphoblastic and myeloid leukaemia [126]. In these studies, different imaging techniques were used to study the biodistribution, tumour uptake and dosimetry of diagnostic and therapeutic radiotracers. Furthermore, ex vivo autoradiography was used to confirm target expression in tumour tissues. Additionally, immunohistochemistry and fluorescent staining were also performed ex vivo to confirm target expression and to determine the therapeutic effect on targeted tissues, including staining for cell proliferation (e.g. Ki67) and DNA-strand breaks (e.g. TUNEL), and assess off-target effects by checking for immune infiltration (e.g. CD3-positive cells). Furthermore, additional analysis can be performed on data collected from therapeutic studies in PDX models by, for example, linking therapy response to different genetic/molecular tumour profiles and possibly identifying biomarkers associated with therapy response.

PDX mouse models have advantages over other mouse models, but there are drawbacks, and a few factors should be taken into account. Firstly, tumours must be xenografted into mice immediately after surgical resection from the patient. In general, PDX lines are maintained in mice since the tumour is transplanted from mice to mice for passaging, which requires many resources, including a great number of animals, materials, funds and properly trained personnel. Efforts have been made to cryopreserve primary patient samples for long-term storage and xenografting at later time points. Unfortunately, this has only been successful for some cancer types [127]. Furthermore, there is an overall low success rate of establishing PDX lines, even without cryopreservation. In a TRT study using subcutaneous NSCLC PDX models, the integrin alpha-V beta-3 (α_v_ß_3_)-targeting radiotracer [^177^Lu]Lu-EB-RGD was evaluated for theranostics in immunodeficient BALB/c nude mice, with successful generation and maintenance of PDX models from only three out of eight tumour samples [125]. The success rate of PDX lines varies and is influenced by numerous factors, including tumour source (e.g. tumour type, composition and location), prior treatments, tissue sampling methods, and the methods used to generate and maintain PDX models (e.g. xenografted tumour volume, xenograft site and mouse strain used) [118]. Logically, PDX models of rare tumours or those that are difficult to establish are under-represented compared to PDX models derived from more common cancer types.

Secondly, the histological, genetic and molecular profile of PDX models should be compared to the original tumour sample regularly when experimental results are linked to specific tumour characteristics. In addition to this, the TME can change overtime, because human stromal cells in the tumour are replaced by murine stromal cells [118], which should also be checked regularly. If such checks are not regularly executed, there is a risk of drawing false conclusions [128].

Lastly, PDX models using immunocompromised mice are still not relevant to study tumour-immune interactions due to the lack of a functional immune system. Moreover, the absence of immune cells also impacts the composition and interactions with(in) the TME [129]. This can be circumvented by using humanized mice.

### Genetically modified mouse models

Genetically engineered mouse models (GEMMs) were established to study the role of individual components in cancer development, progression and therapy response [130]. In general, GEMMs can be divided into models that experience loss of function (i.e. knockout model) or gain of function (i.e. knock-in model). The knockout model is crucial for studying the role of a specific gene in cancer development and its potential impact on therapy response. The ‘knock-in’ model is mainly used to investigate oncogenes related to cancer development. Such ‘knock-in’ models can develop spontaneous tumours as a result of (a) genetic mutation(s). Conventional GEMMs are created by editing the genome of zygotes or embryonic stem cells using a direct locus-specific mutational approach or expression of interfering noncoding RNAs (RNAi) against the target gene [131]. The main limitation of GEMMs is that the number of genes that can be targeted is limited. As such, a single GEMM cannot entirely reproduce the complexity or heterogeneity of most cancers, including extensive aneuploidy and loss- or gain-of-function features.

Traditionally, the establishment of GEMMs is expensive, laborious, and frequently requires extended validation before its implementation because tumour growth is slow and varies among animals [132]. However, the generation of GEMMs has become more efficient and less time-consuming because of recent advances in rapid and efficient sequence-targeted genome editing approaches, including clustered regularly interspaced short palindromic repeats (CRISPR)/Cas9 technology, germline engineering and somatic engineering [133]. Oncogenes and tumour suppressor genes can be directly engineered in specific somatic cells of adult mice using the CRISPR/Cas9 method, which enhances the feasibility of introducing genetic modifications [134]. Editing target genes in adult mice makes this model more representative, as human cancer typically develops later in life. Moreover, the tumour formation in GEMMs is representative of human cancers, as it occurs spontaneously after genetic manipulation of one or more genes in mice with a normal physiology and immune system [135]. This allows an accurate evaluation of the interactions between the tumour and its TME. However, as the tumour still forms in a mouse model, this strategy might not fully recapitulate the role of TME components in human disease progression or therapy response. With genome editing tools like CRISPR/Cas9, it is now possible to induce the expression of human genes in animal cells to generate humanized mouse models, which is specifically useful when designing and studying immunotherapies [136]. The biggest challenge with CRISPR/Cas9 is its off-target effects, as Cas9 can identify sequences with up to five mismatches, which increases the likelihood of off-target effects compared to other genome editing methods [137].

To our knowledge, GEMMs are only sporadically used in the field of TRT. However, GEMMs could be valuable models to study the impact of specific genes on therapeutic efficacy. In line herewith, such models have been applied in other related fields. For example, Wang et al. used conditional knockout mice for YTHDF2 (i.e. a regulator of mRNA stability) that were inoculated with murine colon carcinoma cells to evaluate radiotherapy response [138]. EBRT resulted in pronounced inhibition of tumour growth in Ythdf2-c knock-out mice compared to wild-type mice, assessed by both tumour volume and animal survival [138]. This indicates that inhibition of YTHDF2 could enhance external radiotherapy.

Although GEMMs are an important research tool, each ‘knock-out‘ or ‘knock-in’ model only represents a specific aspect of tumour biology, and thus there is not one model that fully recapitulates a specific cancer type. Furthermore, the murine immune system does not completely reflect that of humans, which could hinder clinical translation of preclinical research. Therefore, more complex and, ideally, more representative models of the human immune system may be required for some cancer studies.

### Humanized mouse models

An alternative to immune incompetent and syngeneic mouse models are humanized mouse models (HMM). The mouse immune system in these models is replaced (“humanized”) by human-derived immune components to generate humanized microenvironments within the mouse that mimic the human immune system. This can be done by engrafting human-derived peripheral blood mononuclear cells (PBMCs), haematopoietic stem cells (HSCs), spleen mononuclear cells or even human foetal bone, liver, or thymus tissue, resulting in a more realistic representation of the human immune system [139]. Since the immune system interacts closely with tumours during disease development and the progression to metastasis, humanized models can be used to investigate the role of the immune system in cancer therapy response. This will provide more realistic and clinically translatable outcomes compared to studies performed in syngeneic mouse tumour models that lack human immune cells.

HMMs are particularly relevant when research areas of immunology and oncology overlap, for example, when studying immunotherapies and their combination with other anti-cancer drugs. In the TRT field, HMMs can be used to investigate the role of the immune system in radiotracer uptake within tumours and immune-related organs, therapy response and potential abscopal effects. Up to now, a few studies using advanced imaging techniques, including SPECT/CT and PET imaging, have used humanized mice to study radiotracers targeting programmed cell death protein 1 (PD-1) expressed by activated T-cells and its ligand programmed death ligand 1 (PD-L1) expressed by tumour cells. However, it should be taken into account that PD-L1 can also be expressed by other immune cells of healthy tissues [140], which could result in high background signals during imaging and impact tumour detection. In a study by Heskamp et al. [^111^In]In-anti-hPD-L1 was used to determine if microSPECT/CT imaging could be used to measure PD-L1 expression in humanized mice bearing subcutaneous MDA-MB-231 human BC tumours [141]. High [^111^In]In-anti-hPD-L1 tumour uptake was observed in both humanized and non-humanized mice, without a significant difference between the two models. The biodistribution of [^111^In]In-anti-hPD-L1 was similar in both models, except for radiotracer accumulation in the spleen and lymph nodes of some humanized mice. Despite the presence of PD-L1 on non-cancerous cells in humanized mice, uptake of [^111^In]In-anti-hPD-L1 by PD-L1-positive tumours was not hindered, and tumours could be visualised successfully using microSPECT/CT imaging [141].

The opposite was observed for the PD-1-targeted radiotracer [^89^Zr]Zr-anti–hPD-1 in a study described by England et al. [142]. Here, [^89^Zr]Zr-anti–hPD-1 was used to visualise the infiltration of PD-1-expressing T-cells in non-humanized and humanized mice bearing subcutaneous A549 lung cancer tumours. The tumour uptake of [^89^Zr]Zr-anti–hPD-1 was higher in humanized mice compared to non-humanized mice from 72 hours onwards due to T-cell infiltration in the tumours of the humanized mice [142]. Additionally, specific salivary gland uptake was observed in humanized mice. The higher uptake of [^89^Zr]Zr-anti–hPD-1 in both the tumour and salivary glands of humanized mice can be explained by lymphocyte infiltration due to “graft versus host disease” (GvHD) in this specific mouse model. Furthermore, ex vivo biodistribution studies showed higher radiotracer uptake in organs of humanized mice, e.g. the liver and spleen. This indicates that manipulation of the immune system can play an important role in PK, biodistribution, and immunogenicity, which can impact therapy response.

While HMMs offer several advantages, they also present certain limitations. HMMs do not fully recapitulate the human immune system, and all other tissues remain entirely murine. Consequently, the TME is composed of a mix of murine cells and human-derived immune cells, which can lead to GvHD, as mentioned above [143]. Approaches to address some of these shortcomings are being investigated, by for example, the development of mouse models expressing human major histocompatibility complex (MHC) genes (i.e. human leukocyte antigens) instead of murine MHC genes. This would limit the rejection of murine tissues and delay the development of lethal human anti-mouse GvHD [144]. So far, the potential impact of a humanized immune system on cell line-derived tumours has not been widely studied, but it has been shown that a humanized TME enhanced both prostate tumour growth and metastasis in orthotopic HMMs [145]. This suggests that tumour biology and therapy response could be significantly affected by the TME in patients.

## Discussion

Each research model described in this review can contribute to the developmental process of novel and/or improved cancer therapies, including TRT. The discussed models all differ in complexity and have specific advantages and limitations, making them suitable for different types of studies. Cancer progression and the development of therapy resistance form a multistage process that is shaped by numerous intrinsic and extrinsic factors occurring simultaneously. However, preclinical models are static and reflect only a specific cancer stage and state, often failing to preserve all cancer characteristics, such as a loss of target expression. This should be considered when designing preclinical studies.

Although 2D cell cultures still form the primary in vitro model for cancer research, the use of 3D cultures, such as spheroids and organoids, has become more common during the development of new therapies. These more complex models are occasionally combined with microfluidic systems to further increase model complexity. An additional benefit of such in vitro 3D models is that it supports the 3Rs principle, which aims to replace, reduce and refine the use of animal models. As the TRT field advances, the need for more complex research models becomes increasingly evident, especially with the recent application of more diverse radionuclides and the development of new therapies focusing on multiple targets (e.g. heterodimers to enhance tumour targeting). For these studies, simple 2D cell cultures are not sufficient anymore.

Unfortunately, to date, the trend of shifting towards more complex in vitro models is not as prominent in the TRT field as in other cancer research fields. For now, mainly spheroids are used to introduce complexity in in vitro preclinical research. Still, other models such as 2D co-cultures and many in vitro 3D models (e.g. organoids and TSCs) have added benefits for TRT studies, for example for studying crossfire, bystander and abscopal effects that are now studied (to a limited extent) using in vivo models.

Concerning in vivo models in the TRT field, cancer cell line-derived tumour models remain the model of choice, despite its limitations and the availability of more advanced models. For example, studies on TRT efficacy in cell line-derived tumour models face limitations because it is extremely challenging to study abscopal effects in these models, where tumours are formed in a non-native microenvironment and are therefore unlikely to metastasize. Moreover, complex models such as HMMs can be used to study the influence of the immune system and the TME on therapy response, which is only partially possible in cell line-derived mouse models. This is particularly important given that abscopal effects and TME components play an important role in cancer treatment response. Furthermore, improvement of preclinical in vivo models is also necessary for the accurate evaluation of off-target organ uptake of radiotracers. For example, almost 39% of the patients treated with [^177^Lu]Lu-PSMA-617 for PCa experience grade 1 or 2 xerostomia [146], due to high off-target radiotracer uptake in the salivary glands, which causes therapy withdrawal by patients. This side effect was unexpected, as salivary gland toxicity was not observed during in vivo studies of [^177^Lu]Lu-PSMA-617 performed in mice, because unlike humans, these animals do not express PSMA in the salivary glands.

On the contrary, for both in vitro and in vivo models, it is important to realise that while more complex research models can be used, the level of complexity should align with the goal and specific research question. For example, there is no need to use valuable organoids for initial affinity and uptake studies of radiotracers, while the same information can be obtained using 2D cell cultures that are less labour intensive and cheaper.

In conclusion, the implementation of other (more complex) preclinical in vitro and in vivo models in TRT research can provide valuable insights that currently used models cannot provide or lack. By carefully selecting preclinical models that mirror clinical conditions, the translation of preclinical research to clinical practice can be improved, which ultimately enhances the development of new or improved targeted radiotracers.

## Key points

### In vitro research models

**2D cell cultures** are widely available and are relevant for high-throughput experiments but lack the complexity to mimic the TME, limiting their relevance for more advanced therapeutic studies.

**Spheroids** can mimic solid tumour features such as hypoxia and nutrient/oxygen gradients that influence therapeutic efficacy, but these models still lack a TME and a representative tumour morphology.

**Organoids** are derived from primary tissues and retain a representative TME, making them relevant for personalised medicine and advanced therapeutic studies, but their variability and limited scalability pose challenges for routine use in research.

**Organotypic tissue slice cultures** maintain original tissue morphology and function and are useful for investigating (therapeutic) interactions in the TME, but these models are complex to maintain and have a short lifespan.

**Microfluidic systems** are highly customisable and promising in determining radiotracer pharmacokinetics and pharmacodynamics, but handling is complex and standardised protocols are not available yet.

### Chorioallantoic membrane model

**Chorioallantoic membrane models** offer rapid tumour development, allowing the investigation of tumour and metastasis formation together with therapy response, but the model is limited to short-term studies due to time constraints, and there is a limited availability of suitable reagents.

### In vivo research models

**Cell line-derived models** are cost-effective and reproducible but often lack tumour complexity and a representative TME.

**Patient-derived xenograft models** retain original tumour morphology and genetics, making them relevant models for studying patient-specific tumour biology and therapy responses, but these models lack a functional immune system and there is a low success rate of tumour engraftment.

**Genetically engineered mouse models** provide valuable insights into the effect of genetic alterations in cancer development, but tumour growth in these models and genetic alteration of a single gene cannot entirely reproduce the complexity and/or heterogeneity of most cancers.

**Humanized mouse models** have functional human immune cells and are relevant for studying the effect of the immune system on therapy, but TME is not fully representative because these models still have murine cells, which can also result in GvHD.

## Data Availability

Not applicable.

## References

[1] Bray F, Laversanne M, Sung H, Ferlay J, Siegel RL, Soerjomataram I, Jemal A. Global cancer statistics 2022: GLOBOCAN estimates of incidence and mortality worldwide for 36 cancers in 185 countries. Cancer J Clin. 2024;74:229–63. 10.3322/caac.21834. 10.3322/caac.21834 38572751

[2] Ferlay J, Colombet M, Soerjomataram I, Parkin DM, Piñeros M, Znaor A, Bray F. Cancer statistics for the year 2020: an overview. Int J Cancer. 2021;149:778–89. 10.1002/ijc.33588. 10.1002/ijc.33588 33818764

[3] Lepareur N, Ramee B, Mougin-Degraef M, Bourgeois M. Clinical advances and perspectives in targeted Radionuclide Therapy. Pharmaceutics. 2023;15. 10.3390/pharmaceutics15061733. 10.3390/pharmaceutics15061733 PMC10303056 37376181

[4] Burkett BJ, Bartlett DJ, McGarrah PW, Lewis AR, Johnson DR, Berberoglu K, et al. A review of Theranostics: perspectives on emerging approaches and clinical advancements. Radiol Imaging Cancer. 2023;5:e220157. 10.1148/rycan.220157. 37477566 10.1148/rycan.220157 PMC10413300

[5] Pouget J-P, Lozza C, Deshayes E, Boudousq V, Navarro-Teulon I. Introduction to radiobiology of targeted radionuclide therapy. Front Med. 2015;2:12. 10.3389/fmed.2015.00012. 10.3389/fmed.2015.00012 PMC4362338 25853132

[6] Aghevlian S, Boyle AJ, Reilly RM. Radioimmunotherapy of cancer with high linear energy transfer (LET) radiation delivered by radionuclides emitting α-particles or Auger electrons. Adv Drug Deliv Rev. 2017;109:102–18. 10.1016/j.addr.2015.12.003. 26705852 10.1016/j.addr.2015.12.003

[7] Widel M. Radionuclides in radiation-induced bystander effect; may it share in radionuclide therapy. Neoplasma. 2017;64:641–54. 10.4149/neo_2017_501. 28592116 10.4149/neo_2017_501

[8] Srinivas US, Tan BWQ, Vellayappan BA, Jeyasekharan AD. ROS and the DNA damage response in cancer. Redox Biol. 2019;25:101084. 10.1016/j.redox.2018.101084. 30612957 10.1016/j.redox.2018.101084 PMC6859528

[9] Park S, Parihar AS, Bodei L, Hope TA, Mallak N, Millo C, et al. Somatostatin Receptor Imaging and theranostics: current practice and future prospects. J Nucl Med. 2021;62:1323–9. 10.2967/jnumed.120.251512. 34301785 10.2967/jnumed.120.251512 PMC9364764

[10] FDA Letter of Approval for Netspot^®^. Available online: https://www.accessdata.fda.gov/drugsatfda_docs/appletter/2016/208547Orig1s000ltr.pdf

[11] FDA Letter of Approval for Lutathera^®^. Available online: https://www.accessdata.fda.gov/drugsatfda_docs/appletter/2018/208700Orig1s000ltr.pdf

[12] FDA Approves First PSMA-Targeted PET Imaging Drug for Men with Prostate Cancer. U.S. Food and Drug Administration. 2020. Available online: https://www.fda.gov/news-events/press-announcements/fda-approves-first-psma-targeted-pet-imaging-drug-men-prostate-cancer

[13] FDA Letter of Approval for Pluvicto^®^. Available online: https://www.accessdata.fda.gov/drugsatfda_docs/appletter/2022/215833Orig1s000ltr.pdf

[14] Fitzgerald AA, Weiner LM. The role of fibroblast activation protein in health and malignancy. Cancer Metastasis Rev. 2020;39:783–803. 10.1007/s10555-020-09909-3. 32601975 10.1007/s10555-020-09909-3 PMC7487063

[15] van der Heide CD, Dalm SU. Radionuclide imaging and therapy directed towards the tumor microenvironment: a multi-cancer approach for personalized medicine. Eur J Nucl Med Mol Imaging. 2022;49:4616–41. 10.1007/s00259-022-05870-1. 35788730 10.1007/s00259-022-05870-1 PMC9606105

[16] Sun J, Huangfu Z, Yang J, Wang G, Hu K, Gao M, Zhong Z. Imaging-guided targeted radionuclide tumor therapy: from concept to clinical translation. Adv Drug Deliv Rev. 2022;114538. 10.1016/j.addr.2022.114538. 10.1016/j.addr.2022.114538 36162696

[17] Dalm SU, Bakker IL, de Blois E, Doeswijk GN, Konijnenberg MW, Orlandi F, et al. 68Ga/177Lu-NeoBOMB1, a novel radiolabeled GRPR antagonist for theranostic use in oncology. J Nucl Med. 2017;58:293–9. 10.2967/jnumed.116.176636. 27609789 10.2967/jnumed.116.176636

[18] Dalm SU, Martens JWM, Sieuwerts AM, van Deurzen CHM, Koelewijn SJ, de Blois E, et al. In vitro and in vivo application of radiolabeled gastrin-releasing peptide receptor ligands in breast cancer. J Nucl Med. 2015;56:752–7. 10.2967/jnumed.114.153023. 25791989 10.2967/jnumed.114.153023

[19] Dalm SU, Sieuwerts AM, Look MP, Melis M, van Deurzen CHM, Foekens JA, et al. Clinical relevance of targeting the gastrin-releasing peptide receptor, somatostatin receptor 2, or chemokine CXC motif receptor 4 in breast cancer for imaging and therapy. J Nucl Med. 2015;56:1487–93. 10.2967/jnumed.115.160739. 26251419 10.2967/jnumed.115.160739

[20] Damiana TST, Paraïso P, de Ridder C, Stuurman D, Seimbille Y, Dalm SU. Side-by-side comparison of the two widely studied GRPR radiotracers, radiolabeled NeoB and RM2, in a preclinical setting. Eur J Nucl Med Mol Imaging. 2023;50:3851–61. 10.1007/s00259-023-06364-4. 37584725 10.1007/s00259-023-06364-4 PMC10611828

[21] Ovchinnikov DA. Genetically-modified cell lines: categorisation and considerations for characterisation. Stem Cell Res. 2020;49:102103. 10.1016/j.scr.2020.102103. 33291011 10.1016/j.scr.2020.102103

[22] Freedman LP, Gibson MC, Ethier SP, Soule HR, Neve RM, Reid YA. Reproducibility: changing the policies and culture of cell line authentication. Nat Methods. 2015;12:493–7. 10.1038/nmeth.3403. 26020501 10.1038/nmeth.3403

[23] Ben-David U, Siranosian B, Ha G, Tang H, Oren Y, Hinohara K, et al. Genetic and transcriptional evolution alters cancer cell line drug response. Nature. 2018;560:325–30. 10.1038/s41586-018-0409-3. 30089904 10.1038/s41586-018-0409-3 PMC6522222

[24] Meijer TG, Naipal KAT, Jager A, van Gent DC. Ex vivo tumor culture systems for functional drug testing and therapy response prediction. Future Sci OA. 2017;3:FSO190. 10.4155/fsoa-2017-0003. 28670477 10.4155/fsoa-2017-0003 PMC5481868

[25] Giard DJ, Aaronson SA, Todaro GJ, Arnstein P, Kersey JH, Dosik H, Parks WP. In vitro cultivation of human tumors: establishment of cell lines derived from a series of solid tumors. J Natl Cancer Inst. 1973;51:1417–23. 10.1093/jnci/51.5.1417. 4357758 10.1093/jnci/51.5.1417

[26] Hofving T, Arvidsson Y, Almobarak B, Inge L, Pfragner R, Persson M, et al. The neuroendocrine phenotype, genomic profile and therapeutic sensitivity of GEPNET cell lines. Endocrine-related Cancer. 2018;25:367–80. 10.1530/ERC-17-0445. 29444910 10.1530/ERC-17-0445 PMC5827037

[27] Klomp MJ, Dalm SU, De Jong M, Feelders RA, Hofland J, Hofland LJ. Epigenetic regulation of somatostatin and somatostatin receptors in neuroendocrine tumors and other types of cancer. Reviews Endocr Metabolic Disorders. 2021;22:495–510. 10.1007/s11154-020-09607-z. 10.1007/s11154-020-09607-z PMC8346415 33085037

[28] Taylor JE, Theveniau MA, Bashirzadeh R, Reisine T, Eden PA. Detection of somatostatin receptor subtype 2 (SSTR2) in established tumors and tumor cell lines: evidence for SSTR2 heterogeneity. Peptides. 1994;15:1229–36. 10.1016/0196-9781(94)90146-5. 7854974 10.1016/0196-9781(94)90146-5

[29] Grozinsky-Glasberg S, Shimon I, Rubinfeld H. The role of cell lines in the study of neuroendocrine tumors. Neuroendocrinology. 2012;96:173–87. 10.1159/000338793. 22538498 10.1159/000338793

[30] Boora GK, Kanwar R, Kulkarni AA, Pleticha J, Ames M, Schroth G, et al. Exome-level comparison of primary well-differentiated neuroendocrine tumors and their cell lines. Cancer Genet. 2015;208:374–81. 10.1016/j.cancergen.2015.04.002. 26087898 10.1016/j.cancergen.2015.04.002

[31] Liu X, Ory V, Chapman S, Yuan H, Albanese C, Kallakury B, et al. ROCK inhibitor and feeder cells induce the conditional reprogramming of epithelial cells. Am J Pathol. 2012;180:599–607. 10.1016/j.ajpath.2011.10.036. 22189618 10.1016/j.ajpath.2011.10.036 PMC3349876

[32] Wu X, Wang S, Li M, Li J, Shen J, Zhao Y, et al. Conditional reprogramming: next generation cell culture. Acta Pharm Sinica B. 2020;10:1360–81. 10.1016/j.apsb.2020.01.011. 10.1016/j.apsb.2020.01.011 PMC7488362 32963937

[33] Ellis L, Ku S, Li Q, Azabdaftari G, Seliski J, Olson B, et al. Generation of a C57BL/6 MYC-driven mouse model and cell line of prostate cancer. Prostate. 2016;76:1192–202. 10.1002/pros.23206. 27225803 10.1002/pros.23206 PMC6123824

[34] Timofeeva OA, Palechor-Ceron N, Li G, Yuan H, Krawczyk E, Zhong X, et al. Conditionally reprogrammed normal and primary tumor prostate epithelial cells: a novel patient-derived cell model for studies of human prostate cancer. Oncotarget. 2017;8:22741. 10.18632/oncotarget.13937. 28009986 10.18632/oncotarget.13937 PMC5410259

[35] Jung M, Kowalczyk K, Hankins R, Bandi G, Kallakury B, Carrasquilla MA, et al. Novel paired normal prostate and prostate cancer model cell systems derived from African American patients. Cancer Res Commun. 2022;2:1617–25. 10.1158/2767-9764.CRC-22-0203. 36970725 10.1158/2767-9764.CRC-22-0203 PMC10035501

[36] Jin L, Qu Y, Gomez LJ, Chung S, Han B, Gao B, et al. Characterization of primary human mammary epithelial cells isolated and propagated by conditional reprogrammed cell culture. Oncotarget. 2018;9:11503. 10.18632/oncotarget.23817. 29545915 10.18632/oncotarget.23817 PMC5837767

[37] Grönman M, Moisio O, Li XG, Toimela T, Huttala O, Heinonen T, et al. Association between [68Ga]NODAGA-RGDyK uptake and dynamics of angiogenesis in a human cell-based 3D model. Mol Biol Rep. 2021;48:5347–53. 10.1007/s11033-021-06513-8. 34213709 10.1007/s11033-021-06513-8 PMC8318966

[38] Van Der Heide CD, Lak A, McMorrow R, Mezzanotte L, Dalm SU. Novel co-culture model of pancreatic Cancer to accurately predict the efficacy of fibroblast activation protein targeted Radionuclide Therapy using [161 tb]Tb- FAP-2286 and [177Lu]Lu-FAP-2286. Eur J Nucl Med Mol Imaging. 2023;50:S187–8. 10.1007/s00259-023-06333-x.

[39] Shi D, Si Z, Cheng Y, Cheng D, Shi H. Synthesis and evaluation of 68Ga-NOTACOG1410 targeting to TREM2 of TAMs as a specific PET probe for Digestive Tumor diagnosis. J Nucl Med. 2023;63. 10.1021/acs.analchem.1c04701. 10.1021/acs.analchem.1c04701 35195007

[40] Privé BM, Boussihmad MA, Timmermans B, van Gemert WA, Peters SMB, Derks YHW, et al. Fibroblast activation protein-targeted radionuclide therapy: background, opportunities, and challenges of first (pre) clinical studies. Eur J Nucl Med Mol Imaging. 2023;50:1906–18. 10.1007/s00259-023-06144-0. 36813980 10.1007/s00259-023-06144-0 PMC10199876

[41] Kapałczyńska M, Kolenda T, Przybyła W, Zajączkowska M, Teresiak A, Filas V, et al. 2D and 3D cell cultures–a comparison of different types of cancer cell cultures. Archives Med Sci. 2018;14:910–9. 10.5114/aoms.2016.63743. 10.5114/aoms.2016.63743 PMC6040128 30002710

[42] Lagies S, Schlimpert M, Neumann S, Wäldin A, Kammerer B, Borner C, Peintner L. Cells grown in three-dimensional spheroids mirror in vivo metabolic response of epithelial cells. Commun Biology. 2020;3:246. 10.1038/s42003-020-0973-6. 10.1038/s42003-020-0973-6 PMC7237469 32427948

[43] Foty R. A simple hanging drop cell culture protocol for generation of 3D spheroids. JoVE (Journal Visualized Experiments). 2011;e2720. . 10.3791/2720 PMC3197119 21587162

[44] Napolitano AP, Dean DM, Man AJ, Youssef J, Ho DN, Rago AP, et al. Scaffold-free three-dimensional cell culture utilizing micromolded nonadhesive hydrogels. Biotechniques. 2007;43:494–500. 10.2144/000112591. 18019341 10.2144/000112591

[45] Costa EC, Gaspar VM, Coutinho P, Correia IJ. Optimization of liquid overlay technique to formulate heterogenic 3D co-cultures models. Biotechnol Bioeng. 2014;111:1672–85. 10.1002/bit.25210. 24615162 10.1002/bit.25210

[46] Brady D, O’Sullivan JM, Prise KM. What is the role of the bystander response in radionuclide therapies? Front Oncol. 2013;3:215. 10.3389/fonc.2013.00215. 23967404 10.3389/fonc.2013.00215 PMC3746502

[47] Grimes DR, Partridge M. A mechanistic investigation of the oxygen fixation hypothesis and oxygen enhancement ratio. Biomed Phys Eng Express. 2015;1:045209. 10.1088/2057-1976/1/4/045209. 26925254 10.1088/2057-1976/1/4/045209 PMC4765087

[48] Jones B. The influence of hypoxia on LET and RBE relationships with implications for ultra-high dose rates and FLASH modelling. Phys Med Biol. 2022;67. 10.1088/1361-6560/ac6ebb. 10.1088/1361-6560/ac6ebb 35545062

[49] Refet-Mollof E, Najyb O, Chermat R, Glory A, Lafontaine J, Wong P, Gervais T. Hypoxic jumbo spheroids On-A-Chip (HOnAChip): insights into treatment efficacy. Cancers. 2021;13:4046. 10.3390/cancers13164046. 34439199 10.3390/cancers13164046 PMC8394550

[50] Sekihara K, Himuro H, Saito N, Ota Y, Kouro T, Kusano Y, et al. Evaluation of X-ray and carbon-ion beam irradiation with chemotherapy for the treatment of cervical adenocarcinoma cells in 2D and 3D cultures. Cancer Cell Int. 2022;22:391. 10.1186/s12935-022-02810-9. 36494817 10.1186/s12935-022-02810-9 PMC9733259

[51] Wang J, Abbas Rizvi SM, Madigan MC, Cozzi PJ, Power CA, Qu CF, et al. Control of prostate cancer spheroid growth using 213Bi-labeled multiple targeted α radioimmunoconjugates. Prostate. 2006;66:1753–67. 10.1002/pros.20502. 16955401 10.1002/pros.20502

[52] Raitanen J, Barta B, Fuchs H, Hacker M, Balber T, Georg D, Mitterhauser M. Radiobiological Assessment of targeted Radionuclide Therapy with [177Lu]Lu-PSMA-I&T in 2D vs. 3D cell culture models. Int J Mol Sci. 2023;24. 10.3390/ijms242317015. 10.3390/ijms242317015 PMC10706939 38069337

[53] Salerno D, Howe A, Bhatavdekar O, Josefsson A, Pacheco-Torres J, Bhujwalla ZM, et al. Two diverse carriers are better than one: a case study in α-particle therapy for prostate specific membrane antigen-expressing prostate cancers. Bioeng Translational Med. 2022;7. 10.1002/btm2.10266. 10.1002/btm2.10266 PMC9115683 35600657

[54] Stenberg VY, Larsen RH, Ma LW, Peng Q, Juzenas P, Bruland ØS, Juzeniene A. Evaluation of the psma-binding ligand212pb-ng001 in multicellular tumour spheroid and mouse models of prostate cancer. Int J Mol Sci. 2021;22. 10.3390/ijms22094815. 10.3390/ijms22094815 PMC8124365 34062920

[55] Tornes AJK, Stenberg VY, Larsen RH, Bruland ØS, Revheim ME, Juzeniene A. Targeted alpha therapy with the 224Ra/212Pb-TCMC-TP-3 dual alpha solution in a multicellular tumor spheroid model of osteosarcoma. Front Med. 2022;9. 10.3389/fmed.2022.1058863. 10.3389/fmed.2022.1058863 PMC9727293 36507500

[56] Mohajershojai T, Jha P, Boström A, Frejd FY, Yazaki PJ, Nestor M. In Vitro characterization of 177Lu-DOTA-M5A anti-carcinoembryonic Antigen Humanized antibody and HSP90 inhibition for Potentiated Radioimmunotherapy of Colorectal Cancer. Front Oncol. 2022;12. 10.3389/fonc.2022.849338. 10.3389/fonc.2022.849338 PMC9010075 35433442

[57] Lundsten S, Berglund H, Jha P, Krona C, Hariri M, Nelander S, et al. P53-mediated radiosensitization of177lu-dotatate in neuroblastoma tumor spheroids. Biomolecules. 2021;11. 10.3390/biom11111695. 10.3390/biom11111695 PMC8615514 34827693

[58] Reuvers TGA, Grandia V, Brandt RMC, Arab M, Maas SLN, Bos EM, Nonnekens J. Investigating the Radiobiological response to peptide receptor Radionuclide Therapy using patient-derived Meningioma Spheroids. Cancers. 2024;16:2515. 10.3390/cancers16142515. 39061156 10.3390/cancers16142515 PMC11275064

[59] Akil H, Quintana M, Raymond JH, Billoux T, Benboubker V, Besse S, et al. Efficacy of targeted radionuclide therapy using [131I]ICF01012 in 3D pigmented BRAF-and NRAS-mutant melanoma models and in vivo NRAS-mutated melanoma. Cancers. 2021;13:1–20. 10.3390/cancers13061421. 10.3390/cancers13061421 PMC8003594 33804655

[60] Lundsten S, Spiegelberg D, Stenerlöw B, Nestor M. The HSP90 inhibitor onalespib potentiates 177Lu–DOTATATE therapy in neuroendocrine tumor cells. Int J Oncol. 2019;55:1287–95. 10.3892/ijo.2019.4888. 31638190 10.3892/ijo.2019.4888 PMC6831206

[61] Żuk M, Podgórski R, Ruszczyńska A, Ciach T, Majkowska-Pilip A, Bilewicz A, Krysiński P. Multifunctional nanoparticles based on Iron oxide and Gold-198 designed for magnetic hyperthermia and Radionuclide Therapy as a potential Tool for Combined HER2-Positive Cancer treatment. Pharmaceutics. 2022;14. 10.3390/pharmaceutics14081680. 10.3390/pharmaceutics14081680 PMC9415738 36015306

[62] Falzone N, Lee BQ, Able S, Malcolm J, Terry S, Alayed Y, Vallis KA. Targeting micrometastases: the effect of heterogeneous radionuclide distribution on tumor control probability. J Nucl Med. 2019;60:250–8. 10.2967/jnumed.117.207308. 10.2967/jnumed.117.207308 PMC6330061 29959216

[63] Marshall SK, Taweesap M, Saelim B, Pachana V, Benlateh N, Sangangam S, et al. Cytotoxicity enhancement in Osteosarcoma with multifunctional I-131 Radiotherapeutic nanoparticles: in Vitro three-dimensional spheroid model and release kinetics modeling. Molecules. 2024;29. 10.3390/molecules29030630. 10.3390/molecules29030630 PMC10856476 38338373

[64] Pekeč T, Venkatachalapathy S, Shim AR, Paysan D, Grzmil M, Schibli R, et al. Detecting radio- and chemoresistant cells in 3D cancer co-cultures using chromatin biomarkers. Sci Rep. 2023;13:20662. 10.1038/s41598-023-47287-2. 38001169 10.1038/s41598-023-47287-2 PMC10673941

[65] Mortensen ACL, Morin E, Brown CJ, Lane DP, Nestor M. Enhancing the therapeutic effects of in vitro targeted radionuclide therapy of 3D multicellular tumor spheroids using the novel stapled MDM2/X-p53 antagonist PM2. EJNMMI Res. 2020;10. 10.1186/s13550-020-0613-7. 10.1186/s13550-020-0613-7 PMC7163001 32300907

[66] Akil H, Rouanet J, Viallard C, Besse S, Auzeloux P, Chezal JM, et al. Targeted Radionuclide Therapy decreases Melanoma Lung Invasion by modifying epithelial-mesenchymal transition-like mechanisms. Translational Oncol. 2019;12:1442–52. 10.1016/j.tranon.2019.07.015. 10.1016/j.tranon.2019.07.015 PMC6704444 31421458

[67] Świerczewska M, Sterzyńska K, Ruciński M, Andrzejewska M, Nowicki M, Januchowski R. The response and resistance to drugs in ovarian cancer cell lines in 2D monolayers and 3D spheroids. Biomed Pharmacother. 2023;165:115152. 10.1016/j.biopha.2023.115152. 37442067 10.1016/j.biopha.2023.115152

[68] Koch J, Moench D, Maass A, Gromoll C, Hehr T, Leibold T, et al. Three dimensional cultivation increases chemo-and radioresistance of colorectal cancer cell lines. PLoS ONE. 2021;16:e0244513. 10.1371/journal.pone.0244513. 33395433 10.1371/journal.pone.0244513 PMC7781370

[69] Yang S, Hu H, Kung H, Zou R, Dai Y, Hu Y, et al. Organoids: the current status and biomedical applications. MedComm. 2023;4:e274. 10.1002/mco2.274. 37215622 10.1002/mco2.274 PMC10192887

[70] Zhao Z, Chen X, Dowbaj AM, Sljukic A, Bratlie K, Lin L, et al. Organoids Nat Reviews Methods Primers. 2022;2:94. 10.1038/s43586-022-00174-y. 10.1038/s43586-022-00174-y PMC10270325 37325195

[71] Kim J, Koo B-K, Knoblich JA. Human organoids: model systems for human biology and medicine. Nat Rev Mol Cell Biol. 2020;21:571–84. 10.1038/s41580-020-0259-3. 32636524 10.1038/s41580-020-0259-3 PMC7339799

[72] Hofer M, Lutolf MP. Engineering organoids. Nat Reviews Mater. 2021;6:402–20. 10.1038/s41578-021-00279-y. 10.1038/s41578-021-00279-y PMC7893133 33623712

[73] Driehuis E, Kretzschmar K, Clevers H. Establishment of patient-derived cancer organoids for drug-screening applications. Nat Protoc. 2020;15:3380–409. 10.1038/s41596-020-0379-4. 32929210 10.1038/s41596-020-0379-4

[74] Shukla HD, Dukic T, Roy S, Bhandary B, Gerry A, Poirier Y, et al. Pancreatic cancer derived 3D organoids as a clinical tool to evaluate the treatment response. Front Oncol. 2023;12:1072774. 10.3389/fonc.2022.1072774. 36713532 10.3389/fonc.2022.1072774 PMC9879007

[75] Lancaster MA, Knoblich JA. Organogenesis in a dish: modeling development and disease using organoid technologies. Science. 2014;345:1247125. 10.1126/science.1247125. 25035496 10.1126/science.1247125

[76] Lucky SS, Law M, Lui MH, Mong J, Shi J, Yu S, et al. Patient-derived nasopharyngeal cancer organoids for disease modeling and radiation dose optimization. Front Oncol. 2021;11:622244. 10.3389/fonc.2021.622244. 33732646 10.3389/fonc.2021.622244 PMC7959730

[77] Lee DW, Choi SY, Kim SY, Kim HJ, Shin D-Y, Shim J, et al. A novel 3D pillar/well array platform using patient-derived head and neck tumor to predict the individual radioresponse. Translational Oncol. 2022;24:101483. 10.1016/j.tranon.2022.101483. 10.1016/j.tranon.2022.101483 PMC9294182 35850059

[78] Kim S-Y, Kim S-M, Lim S, Lee JY, Choi S-J, Yang S-D, et al. Modeling clinical responses to targeted therapies by patient-derived organoids of advanced lung adenocarcinoma. Clin Cancer Res. 2021;27:4397–409. 10.1158/1078-0432.CCR-20-5026. 34083237 10.1158/1078-0432.CCR-20-5026 PMC9401503

[79] Lassche G, van Boxtel W, Aalders TW, van Hooij O, van Engen-van Grunsven ACH, Verhaegh GW, et al. Development and characterization of patient-derived salivary gland cancer organoid cultures. Oral Oncol. 2022;135:106186. 10.1016/j.oraloncology.2022.106186. 36265373 10.1016/j.oraloncology.2022.106186

[80] He X, Jiang Y, Zhang L, Li Y, Hu X, Hua G, et al. Patient-derived organoids as a platform for drug screening in metastatic colorectal cancer. Front Bioeng Biotechnol. 2023;11:1190637. 10.3389/fbioe.2023.1190637. 37284236 10.3389/fbioe.2023.1190637 PMC10239948

[81] Lawlor KT, Vanslambrouck JM, Higgins JW, Chambon A, Bishard K, Arndt D, et al. Cellular extrusion bioprinting improves kidney organoid reproducibility and conformation. Nat Mater. 2021;20:260–71. 10.1038/s41563-020-00853-9. 33230326 10.1038/s41563-020-00853-9 PMC7855371

[82] Meng F, Shen C, Yang L, Ni C, Huang J, Lin K, et al. Mechanical stretching boosts expansion and regeneration of intestinal organoids through fueling stem cell self-renewal. Cell Regeneration. 2022;11:39. 10.1186/s13619-022-00137-4. 36319799 10.1186/s13619-022-00137-4 PMC9626719

[83] Xing F, Liu Y-C, Huang S, Lyu X, Su SM, Chan UI, et al. Accelerating precision anti-cancer therapy by time-lapse and label-free 3D tumor slice culture platform. Theranostics. 2021;11:9415. . 34646378 10.7150/thno.59533 PMC8490519

[84] Suckert T, Rassamegevanon T, Müller J, Dietrich A, Graja A, Reiche M, et al. Applying tissue slice culture in Cancer Research—insights from Preclinical Proton Radiotherapy. Cancers. 2020;12:1589. 10.3390/cancers12061589. 32560230 10.3390/cancers12061589 PMC7352770

[85] Scott SM, Ali Z. Fabrication methods for microfluidic devices: an overview. Micromachines. 2021;12:319. 10.3390/mi12030319. 33803689 10.3390/mi12030319 PMC8002879

[86] Bhatia SN, Ingber DE. Microfluidic organs-on-chips. Nat Biotechnol. 2014;32:760–72. 10.1038/nbt.2989. 25093883 10.1038/nbt.2989

[87] Sin A, Chin KC, Jamil MF, Kostov Y, Rao G, Shuler ML. The design and fabrication of three-chamber microscale cell culture analog devices with integrated dissolved oxygen sensors. Biotechnol Prog. 2004;20:338–45. 10.1021/bp034077d. 14763861 10.1021/bp034077d

[88] McAleer CW, Long CJ, Elbrecht D, Sasserath T, Bridges LR, Rumsey JW, et al. Multi-organ system for the evaluation of efficacy and off-target toxicity of anticancer therapeutics. Sci Transl Med. 2019;11. 10.1126/scitranslmed.aav1386. 10.1126/scitranslmed.aav1386 31217335

[89] Prantil-Baun R, Novak R, Das D, Somayaji MR, Przekwas A, Ingber DE. Physiologically based pharmacokinetic and pharmacodynamic analysis enabled by microfluidically linked organs-on-chips. Annu Rev Pharmacol Toxicol. 2018;58:37–64. 10.1146/annurev-pharmtox-010716-104748. 29309256 10.1146/annurev-pharmtox-010716-104748

[90] Guo Z, Yang CT, Maritz MF, Wu H, Wilson P, Warkiani ME, et al. Validation of a vasculogenesis microfluidic model for radiobiological studies of the human microvasculature. Adv Mater Technol. 2019;4:1800726. 10.1002/admt.201800726.

[91] Leung CM, De Haan P, Ronaldson-Bouchard K, Kim G-A, Ko J, Rho HS, et al. A guide to the organ-on-a-chip. Nat Reviews Methods Primers. 2022;2:33. 10.1038/s43586-022-00118-6.

[92] Keuper-Navis M, Walles M, Poller B, Myszczyszyn A, van der Made TK, Donkers J, et al. The application of organ-on-chip models for the prediction of human pharmacokinetic profiles during drug development. Pharmacol Res. 2023;195:106853. 10.1016/j.phrs.2023.106853. 37473876 10.1016/j.phrs.2023.106853

[93] Wadman M. FDA no longer needs to require animal tests before human drug trials. Science. 2023;379:127–8. 36634170 10.1126/science.adg6276

[94] Dünker N, Jendrossek V. Implementation of the chick chorioallantoic membrane (CAM) model in radiation biology and experimental radiation oncology research. Cancers. 2019;11:1499. 10.3390/cancers11101499. 31591362 10.3390/cancers11101499 PMC6826367

[95] Fischer D, Fluegen G, Garcia P, Ghaffari-Tabrizi-Wizsy N, Gribaldo L, Huang RY-J, et al. The CAM model—Q&A with experts. Cancers. 2022;15:191. 10.3390/cancers15010191. 36612187 10.3390/cancers15010191 PMC9818221

[96] Mapanao AK, Che PP, Sarogni P, Sminia P, Giovannetti E, Voliani V. Tumor grafted–chick chorioallantoic membrane as an alternative model for biological cancer research and conventional/nanomaterial-based theranostics evaluation. Expert Opin Drug Metabolism Toxicol. 2021;17:947–68. 10.1080/17425255.2021.1879047. 10.1080/17425255.2021.1879047 33565346

[97] Schomaecker K, Braun F, Vazquez SM, Marmann V, Fischer T, Endepols H et al. BPD, an AR ligand for prostate carcinoma targeting. J Nucl Med. 2020;61.

[98] Löffler J, Herrmann H, Scheidhauer E, Wirth M, Wasserloos A, Solbach C, et al. Blocking studies to evaluate receptor-specific Radioligand binding in the CAM Model by PET and MR Imaging. Cancers. 2022;14. 10.3390/cancers14163870. 10.3390/cancers14163870 PMC9406147 36010864

[99] Winter G, Koch ABF, Löffler J, Lindén M, Solbach C, Abaei A, et al. Multi-modal pet and mr imaging in the hen’s egg test-chorioallantoic membrane (Het-cam) model for initial in vivo testing of target-specific radioligands. Cancers. 2020;12. 10.3390/cancers12051248. 10.3390/cancers12051248 PMC7281765 32429233

[100] Kalot G, Godard A, Busser B, Pliquett J, Broekgaarden M, Motto-Ros V, et al. Aza-BODIPY: a new vector for enhanced theranostic boron neutron capture therapy applications. Cells. 2020;9:1953. 10.3390/cells9091953. 32854219 10.3390/cells9091953 PMC7565158

[101] Sarogni P, Zamborlin A, Mapanao AK, Logghe T, Brancato L, van Zwol E, et al. Hyperthermia reduces Irradiation-Induced Tumor Repopulation in an in vivo pancreatic carcinoma model. Adv Biol (Weinh). 2023;7:e2200229. 10.1002/adbi.202200229. 36861331 10.1002/adbi.202200229

[102] Kähler J, Hafner S, Popp T, Hermann C, Rump A, Port M, et al. Heterogeneous nuclear ribonucleoprotein K is overexpressed and contributes to radioresistance irrespective of HPV status in head and neck squamous cell carcinoma. Int J Mol Med. 2020;46:1733–42. 10.3892/ijmm.2020.4718. 32901844 10.3892/ijmm.2020.4718 PMC7521550

[103] Ribatti D. The chick embryo chorioallantoic membrane as a model for tumor biology. Exp Cell Res. 2014;328:314–24. 10.1016/j.yexcr.2014.06.010. 24972385 10.1016/j.yexcr.2014.06.010

[104] Mural RJ, Adams MD, Myers EW, Smith HO, Miklos GLG, Wides R, et al. A comparison of whole-genome shotgun-derived mouse chromosome 16 and the human genome. Science. 2002;296:1661–71. 10.1126/science.1069193. 12040188 10.1126/science.1069193

[105] Bryda EC. The mighty mouse: the impact of rodents on advances in biomedical research. Mo Med. 2013;110:207. 23829104 PMC3987984

[106] Ramasawmy R, Johnson SP, Roberts TA, Stuckey DJ, David AL, Pedley RB, et al. Monitoring the growth of an orthotopic tumour xenograft model: multi-modal imaging assessment with benchtop MRI (1T), high-field MRI (9.4 T), ultrasound and bioluminescence. PLoS ONE. 2016;11:e0156162. 10.1371/journal.pone.0156162. 27223614 10.1371/journal.pone.0156162 PMC4880291

[107] Puaux A-L, Ong LC, Jin Y, Teh I, Hong M, Chow PKH, et al. A comparison of imaging techniques to monitor tumor growth and cancer progression in living animals. Int J Mol Imaging. 2011;2011:321538. 10.1155/2011/321538. 22121481 10.1155/2011/321538 PMC3216304

[108] Kim J-B, Urban K, Cochran E, Lee S, Ang A, Rice B, et al. Non-invasive detection of a small number of bioluminescent cancer cells in vivo. PLoS ONE. 2010;5:e9364. 10.1371/journal.pone.0009364. 20186331 10.1371/journal.pone.0009364 PMC2826408

[109] Kleynhans J, Ebenhan T, Cleeren F, Sathekge MM. Can current preclinical strategies for radiopharmaceutical development meet the needs of targeted alpha therapy? Eur J Nucl Med Mol Imaging. 2024:1–16. 10.1007/s00259-024-06719-5 10.1007/s00259-024-06719-5 PMC11139742 38676735

[110] Landgraf M, McGovern JA, Friedl P, Hutmacher DW. Rational design of mouse models for cancer research. Trends Biotechnol. 2018;36:242–51. 10.1016/j.tibtech.2017.12.001. 29310843 10.1016/j.tibtech.2017.12.001

[111] Zhang W, Fan W, Rachagani S, Zhou Z, Lele SM, Batra SK, Garrison JC. Comparative study of subcutaneous and orthotopic mouse models of prostate cancer: vascular perfusion, vasculature density, hypoxic burden and BB2r-targeting efficacy. Sci Rep. 2019;9:11117. 10.1038/s41598-019-47308-z. 31366895 10.1038/s41598-019-47308-z PMC6668441

[112] Sailer V, von Amsberg G, Duensing S, Kirfel J, Lieb V, Metzger E, et al. Experimental in vitro, ex vivo and in vivo models in prostate cancer research. Nat Reviews Urol. 2023;20:158–78. 10.1038/s41585-022-00677-z. 10.1038/s41585-022-00677-z 36451039

[113] De Veirman K, Puttemans J, Krasniqi A, Ertveldt T, Hanssens H, Romao E, et al. CS1-specific single-domain antibodies labeled with Actinium-225 prolong survival and increase CD8 + T cells and PD-L1 expression in multiple myeloma. Oncoimmunology. 2021;10:2000699. 10.1080/2162402X.2021.2000699. 34777918 10.1080/2162402X.2021.2000699 PMC8583167

[114] Puttemans J, Stijlemans B, Keyaerts M, Vander Meeren S, Renmans W, Fostier K, et al. The road to personalized myeloma medicine: patient-specific single-domain antibodies for anti-idiotypic radionuclide therapy. Mol Cancer Ther. 2022;21:159–69. 10.1158/1535-7163.MCT-21-0220. 34667109 10.1158/1535-7163.MCT-21-0220 PMC9398099

[115] Rbah-Vidal L, Vidal A, Billaud EMF, Besse S, Ranchon-Cole I, Mishellany F, et al. Theranostic approach for metastatic pigmented melanoma using ICF15002, a multimodal radiotracer for both PET imaging and targeted radionuclide therapy. Neoplasia. 2017;19:17–27. 10.1016/j.neo.2016.11.001. 27987437 10.1016/j.neo.2016.11.001 PMC5157796

[116] Hernandez R, Grudzinski JJ, Aluicio-Sarduy E, Massey CF, Pinchuk AN, Bitton AN, et al. 177Lu-NM600 targeted radionuclide therapy extends survival in syngeneic murine models of triple-negative breast cancer. J Nucl Med. 2020;61:1187–94. 10.2967/jnumed.119.236265. 31862799 10.2967/jnumed.119.236265 PMC7413241

[117] Chulpanova DS, Kitaeva KV, Rutland CS, Rizvanov AA, Solovyeva VV. Mouse Tumor models for Advanced Cancer Immunotherapy. Int J Mol Sci. 2020;21. 10.3390/ijms21114118. 10.3390/ijms21114118 PMC7312663 32526987

[118] Liu Y, Wu W, Cai C, Zhang H, Shen H, Han Y. Patient-derived xenograft models in cancer therapy: technologies and applications. Signal Transduct Target Therapy. 2023;8:160. 10.1038/s41392-023-01419-2. 10.1038/s41392-023-01419-2 PMC10097874 37045827

[119] Weroha SJ, Becker MA, Enderica-Gonzalez S, Harrington SC, Oberg AL, Maurer MJ, et al. Tumorgrafts as in vivo surrogates for women with ovarian cancer. Clin Cancer Res. 2014;20:1288–97. 10.1158/1078-0432.CCR-13-2611. 24398046 10.1158/1078-0432.CCR-13-2611 PMC3947430

[120] Wang D, Pham NA, Tong J, Sakashita S, Allo G, Kim L, et al. Molecular heterogeneity of non-small cell lung carcinoma patient‐derived xenografts closely reflect their primary tumors. Int J Cancer. 2017;140:662–73. 10.1002/ijc.30472. 27750381 10.1002/ijc.30472

[121] John T, Kohler D, Pintilie M, Yanagawa N, Pham N-A, Li M, et al. The ability to form primary tumor xenografts is predictive of increased risk of disease recurrence in early-stage non–small cell lung cancer. Clin Cancer Res. 2011;17:134–41. 10.1158/1078-0432.CCR-10-2224. 21081655 10.1158/1078-0432.CCR-10-2224

[122] Fichtner I, Slisow W, Gill J, Becker M, Elbe B, Hillebrand T, Bibby M. Anticancer drug response and expression of molecular markers in early-passage xenotransplanted colon carcinomas. Eur J Cancer. 2004;40:298–307. 10.1016/j.ejca.2003.10.011. 14728946 10.1016/j.ejca.2003.10.011

[123] Wang J, Xing B, Liu W, Li J, Wang X, Li J, et al. Molecularly annotation of mouse avatar models derived from patients with colorectal cancer liver metastasis. Theranostics. 2019;9:3485. . 31281492 10.7150/thno.32033 PMC6587174

[124] Ruigrok EAM, van Vliet N, Dalm SU, de Blois E, van Gent DC, Haeck J, et al. Extensive preclinical evaluation of lutetium-177-labeled PSMA-specific tracers for prostate cancer radionuclide therapy. Eur J Nucl Med Mol Imaging. 2021;48:1339–50. 10.1007/s00259-020-05057-6. 33094433 10.1007/s00259-020-05057-6 PMC8113296

[125] Zhao L, Chen H, Guo Z, Fu K, Yao L, Fu L, et al. Targeted radionuclide therapy in patient-derived xenografts using 177Lu-EB-RGD. Mol Cancer Ther. 2020;19:2034–43. 10.1158/1535-7163.MCT-19-1098. 32847972 10.1158/1535-7163.MCT-19-1098 PMC7541553

[126] Habringer S, Lapa C, Herhaus P, Schottelius M, Istvanffy R, Steiger K, et al. Dual targeting of acute leukemia and supporting niche by CXCR4-directed theranostics. Theranostics. 2018;8:369. . 29290814 10.7150/thno.21397 PMC5743554

[127] Ivanics T, Bergquist JR, Liu G, Kim MP, Kang Y, Katz MH, et al. Patient-derived xenograft cryopreservation and reanimation outcomes are dependent on cryoprotectant type. Lab Invest. 2018;98:947–56. 10.1038/s41374-018-0042-7. 29520054 10.1038/s41374-018-0042-7 PMC6072591

[128] Hoge ACH, Getz M, Zimmer A, Ko M, Raz L, Beroukhim R, et al. DNA-based copy number analysis confirms genomic evolution of PDX models. NPJ Precision Oncol. 2022;6:30. 10.1038/s41698-022-00268-6. 10.1038/s41698-022-00268-6 PMC9050710 35484194

[129] Shi J, Li Y, Jia R, Fan X. The fidelity of cancer cells in PDX models: characteristics, mechanism and clinical significance. Int J Cancer. 2020;146:2078–88. 10.1002/ijc.32662. 31479514 10.1002/ijc.32662

[130] Vandamme TF. Use of rodents as models of human diseases. J Pharm Bioallied Sci. 2014;6:2–9. 10.4103/0975-7406.124301. 24459397 10.4103/0975-7406.124301 PMC3895289

[131] Ganguly K, Batra SK, Kaur S. Mouse models of pancreatic cancer: An ever-emerging arm of cancer drug discovery. Animal Models in Cancer Drug Discovery: Elsevier; 2019; pp. 249– 66.

[132] Li Z, Zheng W, Wang H, Cheng Y, Fang Y, Wu F, et al. Application of animal models in cancer research: recent progress and future prospects. Cancer Manage Res. 2021;2455–75. 10.2147/CMAR.S302565. 10.2147/CMAR.S302565 PMC7979343 33758544

[133] Day C-P, Merlino G, Van Dyke T. Preclinical mouse cancer models: a maze of opportunities and challenges. Cell. 2015;163:39–53. 10.1016/j.cell.2015.08.068. 26406370 10.1016/j.cell.2015.08.068 PMC4583714

[134] Sánchez-Rivera FJ, Papagiannakopoulos T, Romero R, Tammela T, Bauer MR, Bhutkar A, et al. Rapid modelling of cooperating genetic events in cancer through somatic genome editing. Nature. 2014;516:428–31. 10.1038/nature13906. 25337879 10.1038/nature13906 PMC4292871

[135] Kersten K, de Visser KE, van Miltenburg MH, Jonkers J. Genetically engineered mouse models in oncology research and cancer medicine. EMBO Mol Med. 2017;9:137–53. 10.15252/emmm.201606857. 28028012 10.15252/emmm.201606857 PMC5286388

[136] Scheer N, Snaith M, Wolf CR, Seibler J. Generation and utility of genetically humanized mouse models. Drug Discovery Today. 2013;18:1200–11. 10.1016/j.drudis.2013.07.007. 23872278 10.1016/j.drudis.2013.07.007

[137] Roy B, Zhao J, Yang C, Luo W, Xiong T, Li Y, et al. CRISPR/Cascade 9-Mediated Genome Editing-challenges and opportunities. Front Genet. 2018;9:240. 10.3389/fgene.2018.00240. 30026755 10.3389/fgene.2018.00240 PMC6042012

[138] Wang L, Dou X, Chen S, Yu X, Huang X, Zhang L, et al. YTHDF2 inhibition potentiates radiotherapy antitumor efficacy. Cancer Cell. 2023;41. 10.1016/j.ccell.2023.04.019. 1294– 308. e8. 10.1016/j.ccell.2023.04.019 PMC10524856 37236197

[139] Cogels MM, Rouas R, Ghanem GE, Martinive P, Awada A, Van Gestel D, Krayem M. Humanized mice as a Valuable Pre-clinical Model for Cancer Immunotherapy Research. Front Oncol. 2021;11:784947. 10.3389/fonc.2021.784947. 34869042 10.3389/fonc.2021.784947 PMC8636317

[140] Beenen AC, Sauerer T, Schaft N, Dörrie J. Beyond cancer: regulation and function of PD-L1 in health and immune-related diseases. Int J Mol Sci. 2022;23:8599. 10.3390/ijms23158599. 35955729 10.3390/ijms23158599 PMC9369208

[141] Heskamp S, Wierstra PJ, Molkenboer-Kuenen JDM, Sandker GW, Thordardottir S, Cany J, et al. PD-L1 microSPECT/CT imaging for longitudinal monitoring of PD-L1 expression in syngeneic and humanized mouse models for cancer. Cancer Immunol Res. 2019;7:150–61. 10.1158/2326-6066.CIR-18-0280. 30459153 10.1158/2326-6066.CIR-18-0280

[142] England CG, Jiang D, Ehlerding EB, Rekoske BT, Ellison PA, Hernandez R, et al. 89 Zr-labeled nivolumab for imaging of T-cell infiltration in a humanized murine model of lung cancer. Eur J Nucl Med Mol Imaging. 2018;45:110–20. 10.1007/s00259-017-3803-4. 28821924 10.1007/s00259-017-3803-4 PMC5700850

[143] Stribbling SM, Beach C, Ryan AJ. Orthotopic and metastatic tumour models in preclinical cancer research. Pharmacol Ther. 2024;108631. 10.1016/j.pharmthera.2024.108631. 10.1016/j.pharmthera.2024.108631 PMC11781865 38467308

[144] Morillon YM, Sabzevari A, Schlom J, Greiner JW. The development of next-generation PBMC humanized mice for preclinical investigation of cancer immunotherapeutic agents. Anticancer Res. 2020;40:5329–41. 10.21873/anticanres.14540. 32988851 10.21873/anticanres.14540 PMC8344070

[145] McGovern JA, Bock N, Shafiee A, Martine LC, Wagner F, Baldwin JG, et al. A humanized orthotopic tumor microenvironment alters the bone metastatic tropism of prostate cancer cells. Commun Biology. 2021;4:1014. 10.1038/s42003-021-02527-x. 10.1038/s42003-021-02527-x PMC8405640 34462519

[146] Sartor O, De Bono J, Chi KN, Fizazi K, Herrmann K, Rahbar K, et al. Lutetium-177–PSMA-617 for metastatic castration-resistant prostate cancer. N Engl J Med. 2021;385:1091–103. 10.1056/NEJMoa2107322. 34161051 10.1056/NEJMoa2107322 PMC8446332

